# Anti-Inflammatory Interleukins in the Pathogenesis of Atherosclerosis

**DOI:** 10.3390/ijms27115030

**Published:** 2026-06-02

**Authors:** Greta Gujytė, Giedrė Rapševičiūtė, Aleksandra Černiakova, Ieva Petrauskaitė, Agnė Liuizė, Aušra Mongirdienė

**Affiliations:** 1 Medical Academy, Lithuanian University of Health Sciences, 50161 Kaunas, Lithuania; greta.gujyte@stud.lsmu.lt (G.G.); giedre.rapseviciute@stud.lsmu.lt (G.R.); aleksandra.cerniakova@stud.lsmu.lt (A.Č.); ieva.petrauskaite@stud.lsmu.lt (I.P.); agne.liuize@stud.lsmu.lt (A.L.); 2 Department of Biochemistry, Lithuanian University of Health Sciences, 44307 Kaunas, Lithuania

**Keywords:** IL-5, IL-10, IL-13, IL-19, IL-35, IL-37, IL-38, IL-1Ra, IL-36Ra, inflammation, atherosclerosis

## Abstract

In recent years, the study of interleukins (ILs), crucial cytokines involved in inflammation, has garnered significant attention within coronary artery disease including atherosclerosis. This review provides a detailed overview of anti-inflammatory ILs, elucidating their functions within the pathogenesis of atherosclerosis. We examine aspects of all the known anti-inflammatory ILs role in atherosclerosis, the direct impact of these ILs on the inflammation; endothelial, smooth vascular cells and macrophage’s function; and their interactions with signaling pathways and molecules. The potential for diagnostic possibilities and targeted drug therapy to modulate anti-inflammatory ILs activity in atherosclerosis was explored. Taken together, findings from recent studies suggest that the main pathways through which ILs exerts its anti-inflammatory effects are: (1) taking part in the regulation of cholesterol transport or oxidised low-density lipoprotein (oxLDL) phagocytosis (IL-1Ra and IL-36Ra—indirectly); (2) affecting different blood cells’ participation in the inflammation (monocytes, lymphocytes, macrophages); (3) taking place in the remodelation of the arterial wall (affecting smooth muscle and endothelium cells). Overall, IL-35, IL-37, and IL-38 appear to be the most promising for modulation of signaling pathways in experimental works and could be investigated as treatment targets. Recombinant IL-10 is investigated in experimental models as therapeutic tool. IL-1Ra is started being translated into clinical practice already. IL-13 and IL-19 are the least studied. It turns out that anti-inflammatory ILs are unlikely to serve as diagnostic markers for atherosclerosis due to their limited specificity and inconsistent associations with disease progression, as well as insufficient validation in large human cohorts. Moreover, key challenges related to delivery, dosing, and safety remain unresolved.

## 1. Introduction

Atherosclerosis is the main cause of cardiovascular diseases (CVD) [1]. It is a chronic inflammatory pathological condition affecting medium and large arteries. Oxidative stress, lipoprotein metabolism and chronic inflammation are the fields of research interest for better understanding of the development and progression of atherosclerosis nowadays. Oxylipins—a diverse family of bioactive lipids are investigated as triggers of the atherosclerotic inflammation too [2]. All these fields are related with secretion of cytokines, what are investigated as possible diagnostic markers for very beginning of atherosclerosis and treatment targets.

All the cytokines are proteins that act in paracrine manner as communication signals among cells of the immune system and other cells and tissues in the body. They are important in immunity, inflammation, tissue development, and repair [2,3]. Cytokines can be produced by every nucleated cell in the human body [4] and are divided into some families: interferons (IFN), ILs, tumor necrosis factors (TNF), colony stimulating factors (CSF), and transforming growth factors (TGF) [2,3]. ILs mediate and regulate inflammation and immune responses. Regulation of inflammation is very important in the development of atherosclerosis. It is known that some ILs promote inflammation, some—inhibits it, and some—can act in both directions.

Focusing on the results obtained in recent years, we aimed to summarize the knowledge about all the known anti-inflammatory interleukins’ relationship with pathogenesis of atherosclerosis: in which cells they are produced, what effectors induced their synthesis and secretion, and what influence do they have on the development of the atherosclerosis. In the article we chose to analyze only anti-inflammatory ILs. This knowledge will bring more understanding in ways for regulation of atherosclerosis development, plaque formation and will allow us to choose which cytokines concentration in the blood could be useful for the diagnostics of the very onset of atherosclerosis and to establish guidelines for further research including treatment targets.

## 2. Search Strategy and Selection Criteria

The relevant articles were searched on PubMed, PubMed Central, Medline, Google Scholar, and ScienceDirect databases. The search encompassed articles published from these databases’ mainly inception from 2015 to 2026 written in English. Where more recent sources were unavailable, several older publications were cited to support specific statements. They constitute 17% of the cited literature (1998–2014 year). The literature search was conducted using the following keywords: interleukins, IL-5, IL-10, IL-13, IL-19, IL-35, IL-37, IL-38, IL-1Ra, and IL-36Ra, atherosclerosis. Inclusion criteria: research and review articles analyzed cellular sources of the anti-inflammatory ILs, receptor complexes, principal downstream signaling cascades, effects on macrophage polarization and endothelial activation, influence on lipid metabolism, foam cell formation, and plaque formation in vitro, animal, or human studies.

## 3. The Group of Anti-Inflammatory ILs

**Figure 1:**
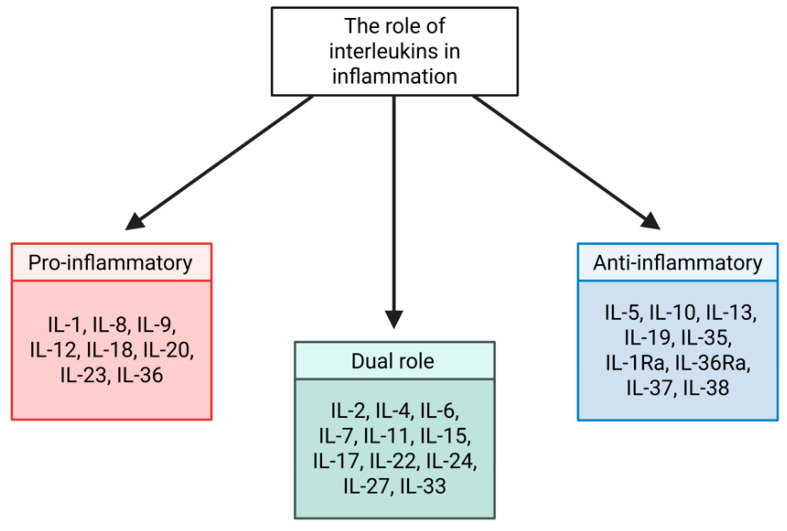
Classification of the interleukins according to the role in inflammation.

Anti-inflammatory ILs (Figure 1) play a crucial role in downregulating immune activation and preventing excessive tissue damage. Since these ILs are known to reduce inflammation, it is suggested that they may also attenuate inflammatory processes within the vascular wall, thereby exerting atheroprotective effects and contributing to the slowing of atherosclerotic plaque progression. Classification of the ILs according to the role in inflammation we managed to find in the literature are given in Figure 1.

### 3.1. Molecular Mechanisms of Anti-Inflammatory ILs on Atherosclerosis Development Pathways

It appears that atherosclerosis can be improved by regulating the Janus kinase/signal transducer and activator of transcription (JAK/STAT) signaling pathway [5]. The JAK family is composed of nonreceptor tyrosine kinases named “Janus kinases” [6]. Janus kinases can phosphorylate both the cytokine receptors, bound to it and multiple specific signaling molecules of the SH2 domain in Signal Transducer and Activator of Transcription (STAT) proteins. The JAK family comprises four members, namely JAK1, JAK2, JAK3, and TYK2 [5]. STAT is a crucial transcription factor in the immune response process. It acts as the downstream target of JAKs and is involved in cellular immunity, apoptosis, and differentiation. The STAT family comprises seven members: STAT1, STAT2, STAT3, STAT4, STAT5A, STAT5B, and STAT6 [7]. STAT contains SH2 and SH3 domains, which can form various heterologous or homologous dimers and bind to specific peptide segments containing phosphorylated tyrosine [8].

**Figure 2:**
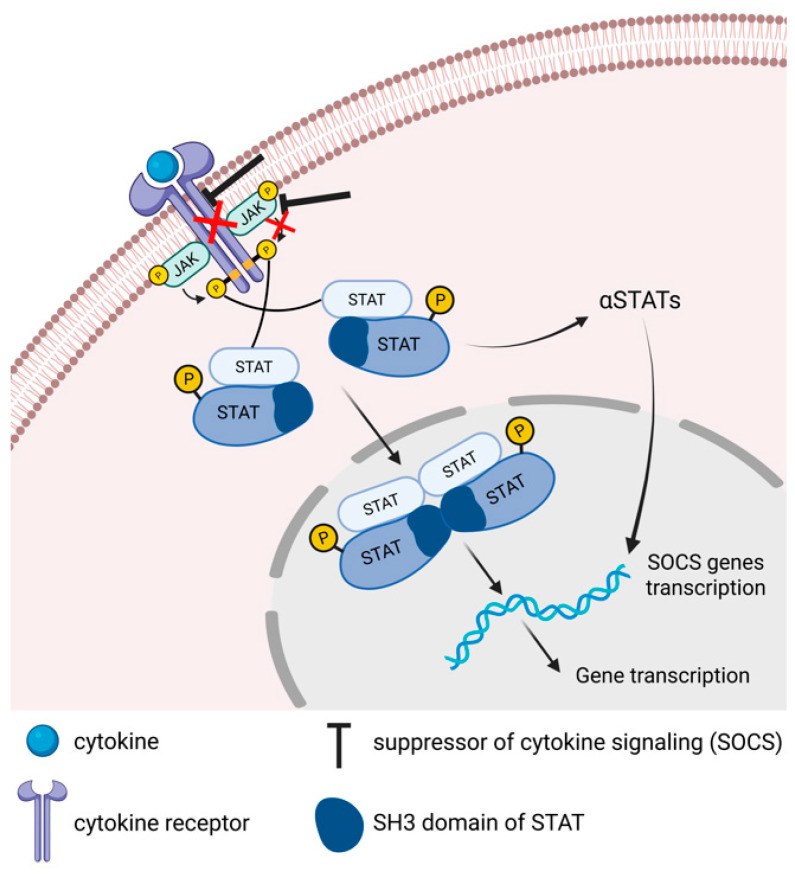
Scheme of Janus kinase/STAT signaling pathway. Binding a cytokine to its receptor on the cell surface induces receptor dimerization and activation of associated Janus kinases (JAKs). Activated JAKs phosphorylate specific tyrosine residues on the receptor cytosolic part, creating docking sites for STAT proteins. STATs are subsequently phosphorylated, dimerized, and translocated into the nucleus, where they bind to DNA and regulate target gene transcription. Suppressor of cytokine signaling (SOCS) stops the signal transduction binding to receptor or JAK. Created using BioRender.

The combination of cytokines and their corresponding receptors can activate signal transduction pathways, transmitting the cytokine signal from the cell membrane to the cytoplasm, and ultimately to the nucleus, resulting in the expression of the target gene (Figure 2). SOCS serves as a negative feedback regulator of the classic JAK/STAT signaling pathway and is also a downstream target gene of this pathway. SOCS can bind to phosphotyrosine on the receptor, preventing the recruitment of STAT to the receptor. It can also directly bind to the JAK receptor, inhibiting JAK activity. Additionally, activated STATs (aSTATs) can stimulate the transcription of SOCS genes [9] (Figure 2). The SOCS family comprises eight proteins, namely SOCS1 to 7 and Cytokine-Inducible SH2-containing protein (CIS) [10]. Among them, SOCS1 and SOCS3 are closely associated with atherosclerosis. These two molecules exhibit anti-inflammatory and protective effects on atherosclerosis in various vascular cells including monocytes, endothelial cells, and smooth vascular muscle cells [11]. JAK2 and STAT3 phosphorylation were regulated in rabbits with atherosclerosis when compared with those of the control group, followed by the expression of SOCS3 was also increased due to the activation of JAK2 and STAT3. Interestingly, ruxolitinib could inactivate JAK2 and STAT3 pathway and decrease SOCS3 expression [12]. It was concluded that the inhibition of JAK2/STAT3/SOCS3 signaling pathway may be a novel method for the clinical treatment of artery atherosclerosis. Despite we aim to summarize recent knowledge about pathways of action of anti-inflammatory ILs related to atherosclerosis development, the extent of available knowledge is dependent on the number of studies conducted and differs across individual ILs.

### 3.2. IL-5

IL-5 is a multifunctional cytokine that stimulates the differentiation of B and T cells [13]. Recent research has revealed that activation of type 2 innate lymphoid cells (ILC2) is associated with lower atherosclerotic burden [14]. It could be related to IL-5 secretion responding to IL-33 and IL-25 stimulation in these cells [14]. Authors point out three mechanisms by which ILC2s may reduce atherosclerosis: (i) the IL-33/IL-25/TSLP-ILC2-IL-5-IgM axis (TSLP—Thymic Stromal Lymphopoietin, ILC2—Group 2 Innate Lymphoid Cells, IgM—Immunoglobulin M); (ii) modulation of lipid homeostasis and (iii) modulation of lesional macrophages [14].

Some studies have suggested that IL-5 levels may be associated with plasma levels of antibodies against oxLDL and that this association may be linked to a reduction in atherosclerosis. These antibodies are known to play an important role in the recognition and removal of oxLDL, possibly reducing the progression of atherosclerotic plaques [15]. Accordingly, it was found that IL-5 is involved in the lipid metabolism of immune cells and that IL-5 acts through IL-5R receptors [13] and stimulates ATP binding cassette transporter A1 (ABCA1) expression and cholesterol efflux in human acute monocytic leukemia cell line THP-1-derived macrophages [16]. ABCA1 plays a significant role in high density lipoprotein (HDL) metabolism whereas cholesterol efflux through ABCA1 mediates the cellular efflux of phospholipids and cholesterol to lipid-poor HDL [17]. Cholesterol accumulation in macrophage foam cells during atherogenesis induces inflammatory responses. Inflammation suppresses cholesterol efflux by suppressing ABCA1 and ATP binding cassette transporter G1 (ABCG1) in macrophages, which in turn enhances inflammation [18]. ABCA1 and ABCG1 suppress macrophage inflammation by disturbing the raft domains where proteins, such as Toll-like receptors, are involved in inflammatory response function [19]. Overexpression of either ABCA1 or ABCG1 reduces inflammatory responses [20]. IL-5 increased ABCA1-mediated cholesterol efflux through the miR-211/JAK2/STAT3 signaling pathway in THP-1-derived macrophages [21]. A compound that activates ABCA1 and ABCG1 is expected to decrease cholesterol accumulation and inflammation in cells and could be effective in suppressing atherosclerosis. So, IL-5 levels may help to reduce macrophage-mediated inflammation in atherosclerotic plaques by increasing ABCA1 expression.

It was shown that IL-5 may limit the apoptosis of smooth muscle cells in the arterial walls too. In coronary artery disease, smooth muscle cells in the arterial wall undergo changes and apoptosis which contribute to plaque instability. Thus, if IL-5 can effectively inhibit this apoptosis, it may also help maintain the stability of atherosclerotic plaques [22].

Recent findings of IL-5 in atherosclerosis development are given in Table 1. The results strongly suggest an atheroprotective role of IL-5. It is established already that IL-5 (1) is involved in cholesterol efflux from the macrophages, (2) take place in reduction of macrophage-mediated inflammation in atherosclerotic plaques, (3) IL-5 may limit apoptosis of smooth muscle cells in the arterial wall. But some data is obtained from people with comorbidities, and. molecular pathways of these actions are not well revealed yet.

### 3.3. IL-10

IL-10 is produced mainly by monocytes but can also be secreted by mast cells to reduce leukocyte infiltration and inflammation [17,27]. IL-10 acts through IL-10R1 and IL-10R2 [28]. It can inhibit the expression of many pro-inflammatory cytokines, chemokines and chemokine receptors. In the context of atherosclerosis, its main roles include inhibition of macrophage activation, matrix metalloproteinase, and expression of pro-inflammatory cytokines and cyclooxygenase-2 in lipid-rich and macrophage-activated fat cells [17,21]. There is evidence that IL-10 plays a protective role against atherosclerosis and thrombosis through its effects on lipoproteins metabolism and vascular inflammation. Several studies have advanced our understanding of how IL10 exerts antiatherogenic effects at the molecular level. Key findings include knowledge that IL-10: (1) protects from atherosclerosis by reducing tumor necrosis factor alpha (TNFα), C-C motif chemokine ligand 2 (CCL2). And Matrix metalloproteinase-9 (MMP-9); (2) decreased macrophage activation; (3) molecular mechanism of action goes through IL10R1/IL10R2/JAK1/tyrosine kinase 2 (TYK2)/phosphorylation of STAT3/transcription of anti-inflammatory genes and suppression proinflammatory cytokines.

Deleting IL10 specifically in myeloid cells, particularly macrophages, in *ApoE*^−/−^ mice leads to significantly increased plaque burden. In these mice, loss of macrophage IL10 results in higher systemic levels of proinflammatory cytokines such as TNFα and chemokines like CCL2, and enhanced T helper 17 cells (Th17) responses [29]. Additionally, the ability of IL-10 to inhibit TNF-αproduction in lipopolysaccharide-stimulated macrophages requires the presence of Stat3, Jak1, and two distinct regions of the IL-10 receptor intracellular domain [30]. Two redundant Stat3 recruitment sites (427YQKQ430 and 477YLKQ480) were required for all IL-10-dependent effects on either B cells or macrophages [31]. Additionally, macrophages’ overexpressed IL10 reduce plaque size and necrotic core.

In a 2024 experiment, RAW264.7 macrophages were engineered to overexpress IL10 (called IL10M), then introduced into *ApoE*^−/−^ mice. These IL10 high macrophages targeted atherosclerotic plaques, reduced plaque area and the necrotic core, decreased expression of MMP9 (involved in plaque rupture), lowered reactive oxygen species, improved mitochondrial membrane potential, and shifted macrophage polarization towards a more anti-inflammatory profile [32]. One more study in mice has shown that IL-10 deficiency is associated with a redistribution of MMP with increased activity and MMP 9 antigen levels, as shown by immunohistological studies of aortic plaques [33]. Other researchers have also suggested that IL-10 inhibits the production of pro-inflammatory cytokines, TNF-α and reduces MMP-9 activity and inflammatory cell infiltration [27,34]. This research indicates that macrophage secreted IL10 plays a suppressive role in inflammation in the plaque environment.

A strategy to fuse IL-10 to a low-density lipoprotein (LDL) binding antibody fragment (FabIL10) allows IL-10 to “hitchhike” on circulating LDL particles and accumulate more in plaques. One such fusion protein (2D03IL10) in murine atherosclerosis models reduced immune cell infiltration into the aorta, particularly macrophages, and decreased expression of proinflammatory markers. In contrast, nontargeted IL-10 had much less effect. This suggests that enhancing localization of IL-10 to plaques improves its molecular impact. The mechanism involves preserved signaling via STAT3, suppression of inflammatory gene expression in macrophages, and decreased macrophage activation [35].

Additionally, IL-10 may inhibit the antigen-presenting function of dendritic cells and inhibit macrophage activation and infiltration into the lesion, thereby reducing their inflammatory behavior and limiting the progression of atherosclerotic plaques [34]. Thus, IL-10 has been shown to inhibit the development and progression of atherosclerosis. Furthermore, IL-10 can reduce oxidative inflammatory products in the vascular wall, as well as the complexity and number of atherosclerotic lesions, plaque size, mast cell apoptosis and IFN-γ expression in activated macrophages (Table 2).

Recently, even the therapeutic effects of IL-10 on atherosclerosis have also been investigated and well discussed by E.H. Steen with co-authors [34]. In short, IL-10 has been a focus of potential antifibrotic therapies because of its role as an anti-inflammatory mediator. More recently, many groups have worked to develop novel delivery tools for recombinant IL-10, such as hydrogels, and cell-based therapies, such as ex vivo activated macrophages, to directly or indirectly modulate IL-10 signaling [34]. All the studies are in progress. The research results confirm the protective role of IL-10 in all the stages of atherogenesis [27] and encourage further investigation of the important therapeutic role of IL-10.

### 3.4. IL-13

IL-13 is a cytokine produced by T_H_2, T, CD4, mast cells, natural killers (NK), basophils and eosinophils [21,41]. It was shown earlier that IL-13 plays an atheroprotective role during atherogenesis: IL-13 can modulate the macrophage phenotype by inducing macrophage polarization towards M2 phenotype, which is associated with anti-inflammatory functions and could potentially help to resolve inflammation and clear cellular debris in the atherosclerotic plaque [42]. IL-13 can act through two types of receptors:/IL-13Ra1 and IL-13Ra2 [43]. Signaling via the IL-13Ra2 has been shown to induce TGF-b1 production in macrophages via a STAT6-independent pathway, leading to collagen deposition in vivo [43]. The later results showed that IL-13-stimulated macrophages have a better uptake of oxLDL. This protective effect of this interleukin is explained by the fact that the increased ability of IL-13-stimulated M2 macrophages to clear oxLDL from the extracellular medium also effectively promotes the efflux of free cholesterol via the ABCA1/G1 pathway, without increasing the formation of adipose cells [43]. IL-13 plays an important role in inhibiting the progression of atherogenesis and promoting plaque stabilization by reducing Vascular Cell Adhesion Molecule (VCAM-1)-mediated monocyte recruitment to atherosclerotic lesions and increasing collagen deposition [42]. Additionally, increased intima-media thickness is associated with lower circulating IL-13 [42]. IL-13 was found to be increased in human asymptomatic plaques [44]. IL-13 can target the type II receptor which shares the IL-4Rα subunit and consists of the additional IL-13Rα1 chain [45] resulting in the inhibition of inflammatory signaling (Table 3).

It is interesting that IL-13 upregulates M2 marker expression in human primary macrophages, STAT3, but not STAT1 or STAT6 inhibition, attenuates IL-13-induced expression of M2 macrophage markers, elevated superoxide production. The peak superoxide production induced by IL-13 was significantly less than that of IL-4, by 17% (* *p* = 0.04) in macrophages [42]. It is thought that some of the protective actions of IL-13 in cardiovascular disease could be mediated via the IL-13Rα2 receptor, leading to increased collagen deposition and plaque stability, or reparative connective tissue formation following MI. Both IL-4 and Il-13 act through the same receptors [42], but to date, no study has directly compared the effects of single cytokine IL-13 or IL-4 deficiency, or exogenous treatment, in the same cohort of diseased animals or humans. Such an approach, in addition to comparing the effects of IL-4 and IL-13 on vascular cells, is required to provide definitive evidence for differential effects of these cytokines in a whole-body system.

Summarizing knowledge about Il-13 could be stated that IL-13 reduces atherosclerosis development (1) inducing macrophage polarization to anti-inflammatory phenotype and better uptake oxLDL, (2) promote plaque stabilization by reducing VCAM-1-mediated monocyte recruitment to atherosclerotic lesions, (3) taking place in increasing collagen deposition and plaque stability. These protection actions could be implemented acting through the IL-13Rα2 receptors. Studies on the role of IL-13 in atherosclerosis are ongoing, and its precise effects are still under active investigation.

### 3.5. IL-19

IL-19 is expressed in multiple cell types within plaques but not in normal arteries, supporting a “lesion-induced” up-regulation of IL-19 [47]. Monocytes, endothelial cells, fibroblasts, and CD8^+^ T cells are the main producers of IL-19 [48]. Accordingly, IL-19 is released from lipopolysaccharide (LPS)-stimulated monocytes, keratinocytes, endothelial, epithelial cells and can be secreted in small amounts by B cells [27,41]. The IL-20R1 and IL-20R2 subunits together form a receptor complex via which IL-19 functions [48]. IL-19 is one of the most significant anti-inflammatory cytokines contributing to plaque healing. IL-19 is presented as a multifunctional cytokine with pleiotropic atheroprotective actions, including immune modulation (Th2 skewing, M2 macrophage polarization), vascular cell regulation (EC/VSMC anti-inflammatory and antiproliferative effects), and macrophage lipid-metabolism control (cholesterol uptake and efflux) [47]. Additionally, genetic deletion of IL-19 in mice (Il19^−/−^ × Ldlr^−/−^) resulted in increased atherosclerotic plaque burden compared with Ldlr-knockout alone, implicating IL-19 as a protective factor [49]. Moreover, IL-19 works to reduce the size of atherosclerotic plaques and has a modest effect on lipid lowering [27]. Data suggest that IL-19 increases lymphatic vessel formation, potentially allowing RCT to take place to decrease plaque burden in atherosclerosis [50]. The activation of VSMCs and the generation of pro-inflammatory molecules including TNF-α, IL-1β, and MCP-1 are caused by IL-19 deficiency [48].

Fujimoto with coauthors reviewed IL-19 as a pleiotropic member of the IL-10 family that modulates immune responses by promoting Th2 polarization, inducing anti-inflammatory cytokines, and suppressing pro-inflammatory mediators in macrophages, endothelial, and smooth muscle cells. It highlights IL-19’s ability to signal mainly through the IL-20Rα/IL-20Rβ receptor complex, activating JAK-STAT3/STAT6 pathways that mediate its anti-inflammatory and tissue-protective effects [51]. However, IL-19’s specific downstream molecular mechanisms in vascular cells, and how these pathways translate into atheroprotection in vivo, remain poorly defined (Table 4). Future studies are needed to clarify how IL-19 integrates lipid metabolism, oxidative stress, and immune cell polarization in the atherosclerotic microenvironment acts, confirm IL-19’s mechanistic pathway in cells and determine its receptor specificity and therapeutic potential in human disease.

### 3.6. IL-35

IL-35 is a member of the IL-12 family, composed of the IL-12p35 and EBV-inducible gene 3 (EBI3) subunits that are induced under inflammatory conditions and are present in the early atherosclerosis development [55]. IL-35 is mainly secreted by regulatory T and B cells, but can also be produced by monocytes, vascular endothelial cells, smooth muscle cells and epithelial cells. IL-35 acts through Il35a and Ebi3 receptor subunits [56]. IL-35 receptor subunit IL35rb2 was highly induced when compared to the IL-35 itself, indicating that the atherosclerotic tissue is a target rather than a source of IL-35 during early atherosclerosis development [57]. It was identified a novel mitochondrial reactive oxygen species (mtROS)-driven site-specific histone 3 lysine 14 (H3K14) acetylation mechanism in driving endothelial cell (EC) activation gene transcription, which could be suppressed by IL-35. In addition, IL-35 selectively inhibits lysophosphatidylcholine (LPC)-induced EC activation-related genes such as intercellular adhesion molecule-1 (ICAM-1) [57]. IL-35. has been shown to have anti-inflammatory effects on regulatory T cells (Tregs) and regulatory B cells (Bregs). IL-35 can inhibit Th17 cell differentiation and increase the suppressive capacity of anti-inflammatory regulatory T cells (Tregs), thereby suppressing the immune response and inflammation and may help to mitigate the inflammatory processes associated with atherosclerosis [58]. It was revealed that IL-35 suppresses the development of atherosclerosis by suppressing EC activation and promoting C-C motif chemokine receptor 5 (CCR5) expression in the Tregs (increased CCR5 enhance the immunosuppressive function of splenic Tregs) too [55]. These effects occur when IL-35 binding to a receptor complex composed of IL-12Rβ2 and gp130 [56].

In research with cell cultures, it was shown that in macrophages stimulated with oxidized low-density lipoprotein (ox-LDL), IL-35 reduces lipid accumulation and foam cell formation. Moreover, IL35 regulates microRNAs associated with coronary artery disease, influencing macrophage polarization, T cell responses, lipoprotein metabolism, and inducible nitric oxide synthase–mediated inflammatory signaling (Table 5) [59]. In human vascular EC, IL35 is upregulated under pro-atherogenic stress, indicating a response to lipid-induced oxidative stress. Lipid-lowering treatment, including rosuvastatin, reduce IL35 subunit expression, highlighting its role in modulating endothelial inflammation [60]. IL-35 further protects endothelial function in human aortic (HAECs) and umbilical vein (HUVECs) endothelial cells by reducing mitochondrial reactive oxygen species (mtROS), limiting activator protein 1 (AP-1) mediated intercellular adhesion molecule 1 (ICAM-1) expression, and decreasing monocyte adhesion. It also modulates proliferation, migration, adhesion, and angiogenesis through signal transducer and activator of transcription 1 (STAT1), STAT3, and STAT4 pathways, mitigating endothelial dysfunction under hypoxia and inflammatory cytokines [61]. IL-35 could significantly inhibit the expression of 10 genes, including insulin like growth factor binding protein 5 (IGFBP5), calpain 3 (CAPN3), tumor necrosis factor receptor associated factor 1 (TRAF1), CMT1A duplicated region transcript 4 (CDRT4), IL1B, TGF-β3 (TGFB3), NF-kB subunit RELB, chemokine (C-X-C motif) ligand 1 (CXCL1), intercellular adhesion molecule 1 (ICAM1), and C-X-C motif chemokine ligand 8 (CXCL8) [57]. These findings indicate that IL-35 acts as an immunoregulatory cytokine at the cellular level, controlling both lipid-induced dysfunction and inflammation in macrophages and endothelial cells.

In mice models of atherosclerosis, treatment with IL-35 reduced the size of atherosclerotic plaques and decreased inflammation in the vessel wall. In models of myocardial injury, such as myocardial infarction and viral myocarditis, IL-35 reduced heart inflammation, limited cell death, and improved tissue repair (Table 2). However, the precise mechanisms by which IL-35 modulate endothelial function, foam cell formation, and macrophage polarization in atherosclerotic lesions remain unclear jet. The roles of its subunits EBI3 and p35, the regulation of IL-35 expression under pro-atherogenic stimuli, require further investigation.

Genetic variations in the IL-12/IL-35 pathway, such as IL12A/p35 variants, in humans was found may influence lipid metabolism and contribute to a pro-atherogenic environment. These variants could affect IL-35 production, alter immune regulation, and modulate inflammatory responses in the vasculature [62], but the exact mechanisms linking genetic differences to cardiovascular risk and IL-35 function remain too (Table 2).

Researchers found that IL-35 concentrations were significantly higher in patients with coronary artery disease (CAD) compared with healthy controls [63]. Accordingly, the levels of IL-35 are significantly induced in hypercholesterolemic patients; and that increased IL-35 levels are positively correlated with cholesterol concentrations [57]. These findings indicate that IL-35 is involved in the immunological processes accompanying CVD in humans and may serve as a potential biomarker of immune activation or regulation in atherosclerosis (Table 2). It should be mentioned that a recent report on CANTOS trial with anti-IL-1β antibody Canakinumab stated IL-35 as a feasible approach in the treatment of cardiovascular diseases [64]. However, further studies are required to determine whether increased IL-35 levels contribute to disease progression, reflect disease severity, or represent a protective regulatory response. It should be clarified how do genetic variants influence IL35 production, lipid metabolism, and plaque development too.

**Table 5:** Main findings of IL-35 in cell culture, animal and human studies.

Found Peculiarities of IL-35	Cell Type/Animal/Humans	References
Exogenous IL-35 decreases lipid accumulation, reducing foam cell formation; has inhibitory effect on ox-LDL induced cell enlargement.	Macrophages (ox-LDL-stimulated).	[59]
IL35 subunits (EBI3, IL12A) increase in endothelial cells under pro-atherogenic lipid stress (25OHC) and decrease after rosuvastatin; indicating that IL35 reacts to lipid stress and reduces inflammation.	Human vascular endothelial cells (HUVEC, in vitro).	[60]
IL-35 suppresses lipid-induced endothelial activation, reduces ICAM-1 expression, and prevents monocyte adhesion.	Human aortic endothelial cells (HAECs).	[61]
IL-35 pathway genetic variant rs2243115 (IL12A/p35) is associated with lower HDL and higher LDL cholesterol, suggesting that IL-35 pathway genetic differences may promote a pro-atherogenic environment, increasing the risk of CAD.	768 patients with CAD, 768 controls.	[62]
Serum IL-35 levels are increased in patients with cardiovascular diseases compared with healthy controls.	50 patients with heart and vascular diseases (atherosclerosis, coronary artery disease). and 25 controls.	[63]
Exogenous IL-35 reduces atherosclerotic lesion formation, decreases aortic intimal thickness and plaque area. IL-35 restores Th17/Treg balance, reducing atherosclerosis progression.	*ApoE*^−/−^ mice (atherosclerotic).	[65]
IL-35 overexpression reduces Th17-related pro-inflammatory cytokines, protecting myocardium from viral myocarditis.	BALB/c male mice infected with Coxsackievirus B3.	
IL-35 improves cardiac repair after myocardial infarction by promoting survival of reparative CX3CR1 + LY6Clow macrophages.	Mouse MI model.	
IL-35 protects cardiomyocytes from mitochondrial ROS-induced apoptosis in myocardial ischemia/reperfusion injury (MIRI).	Newborn mice hypoxia/reoxygenation mode.	

Abbreviations: ox-LDL—oxidized low-density lipoprotein, 25-OHC—25-hydroxycholesterol, ICAM-1– including intercellular adhesion molecule 1, EBI3—Epstein-Barr virus induced gene 3, IL-12A—interleukin-12 subunit alpha.

### 3.7. IL-37

IL-37 is a member of IL-1 family. It can be produced by a wide range of tissues and cells: monocytes, macrophages, dendritic cells, epithelial and endothelial cells, fibroblasts, smooth muscle cells, and has been shown to be produced by certain tumor cells (breast carcinoma, some colon carcinoma, melanoma, lung carcinoma) [41,66] (Table 6). IL-37 seems to have broader anti-inflammatory properties compared to the other members of the IL-1 family. The pathways of IL-37 action on pathogenesis of atherosclerosis are well discussed by Sara McCurdy and coauthors [66,67]. In brief, rs3811047 polymorphism establishes IL-37 as a susceptibility gene for CAD. Each of the 4 IL-37 sequence variations were found in exon 5, encoding an important functional domain of the protein, and the resulting IL-37 proteins were shown to have reduced anti-inflammatory activity in vitro. Results of IL-37 role on monocyte/macrophage activation in vitro experiments differed. Reducing inflammation at the endothelial monolayer by IL-37 has been obtained. The role of IL-37 in coronary artery calcification aspect of CVD remains unclear [67]. Accordingly, the action of IL-37 is thought to involve intracellular and extracellular pathways involving the transcription factor Smad3 and the orphan receptor IL-1R8 respectively in both human THP-1, and mouse RAW264.7 macrophages in vitro [66,67]. The role of Smad3 and IL-37 signaling in atherosclerosis has not yet been investigated. Authors concluded that there is need to a deeper understanding of its role in various cardiovascular pathologies as well as ways to manipulate and administer the cytokine as a potential therapeutic agent against CVD.

IL-37 can inhibit T lymphocyte production of pro-inflammatory cytokines including IL-6, TNF-α and interleukin-1β (IL-1β).

**Figure 3:**
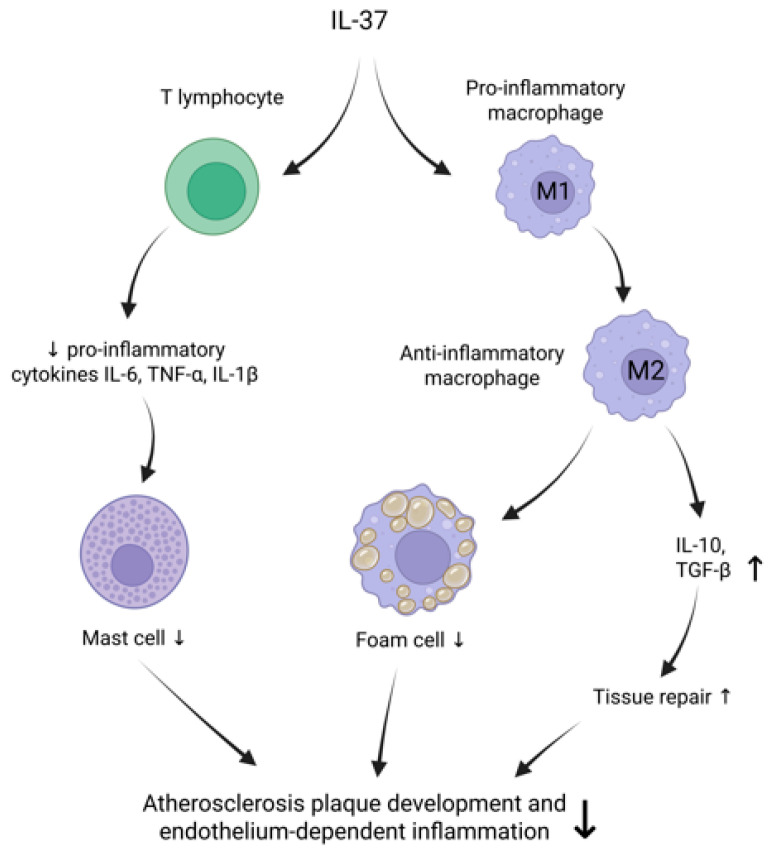
IL-37 role in the development of atherosclerosis. Created using BioRender.

The recent scientific studies show that IL-37 may modulate several pathways in the development of atherosclerosis, including inflammation, macrophage lipid homeostasis and cell survival [66,68,69,70] (Figure 3). Furthermore, IL-37 may attenuate macrophage activation, thereby reducing their ability to promote inflammation and tissue damage in the arterial wall [69]. By modulating these macrophage functions, IL-37 may have a protective effect against atherosclerosis by reducing inflammation, limiting mast cell formation, promoting a more favorable macrophage phenotype, and generally influencing the progression of atherosclerotic plaques to a more stable state [68,69]. In addition, IL-37 can inhibit immune cell activation and modulate the immune response. This modulation may influence atherosclerotic plaque development by reducing the infiltration of inflammatory cells into the vascular wall and by modifying the activity of immune cells that contribute to plaque formation [66,70]. IL-37 is thought to help preserve endothelial function and reduce endothelial inflammation, possibly by preventing the development of atherosclerotic lesions (Figure 3). One study investigated the inhibitory effects of IL-37 on key unfolded protein response (UPR) sensors: PERK (protein kinase RNA- like Endoplasmic Reticulum (ER) kinase), IRE1 (inositol-requiring enzyme-1), and ATF6 (activating transcription factor-6). These sensors have C-termini in the cytosol and N-termini that interact with the ER chaperone GRP78 (glucose-regulated protein 78) located in the lumen of the ER under normal, unstressed conditions. Clearly, IL-37 reduces the expression of all three sensors as well as GRP78, suggesting a potential impact on the development of atherosclerosis [71]. These findings provided a basis for the use of IL-37 as a therapeutic target. But it should be kept in mind that the number of studies examining the modulation of ER stress through this interaction remains limited (Table 6). So, further research is needed to gain a deeper understanding of the interplay between ER stress and autophagy.

Accordingly, studies of recent years in humans confirmed that IL-37 levels, oxidative stress, and the progression of the disease are significantly correlated, and the serum level of IL-37 was lower in CAD patients in comparison with controls [72,73] (Table 6). But to reach more substantial conclusions, studies with larger sample sizes are needed.

**Table 6:** Findings of IL-37 role in atherosclerosis.

Found Peculiarities of IL-37	Studied Object	n (Humans)	References
IL-37 reduces the ox-LDL-induced pro-osteogenic response, ERS, and autophagy by binding to Smad3.	RCAECs		[71]
Serum level of IL-37 was higher in control group, and reversibly correlated with TNF-α, MDA and oxLDL.	CAD/healthy	50/50	[72]
Serum level of IL-37 was higher in control group. IL-37 significantly had an inverse correlation with IL-6, tumor necrosis factor-α, IL-32, high-sensitivity C reactive protein, oxidized low-density lipoprotein, and malondialdehyde. IL-37 had a significantly positive correlation with ATP-binding cassette transporter A1 and G1 gene expression in peripheral blood mononuclear cells and serum levels of the FRAP. IL-37 level ratios were a relatively significant CAD predictor.	CAD/healthy	42/42	[73]
IL-37 was increased in patients with chronic lower limb atherosclerotic ischemia, compared to non-atherosclerotic controls. In addition, the expression levels of circulating IL-37 correlated with disease severity of chronic lower limb ischemia. Supplementation with rIL-37 augmented levels of released IL-10 and TGF-β in supernatants of T cells co-cultured with Tregs in the enrolled patients.	Patients with chronic lower limb atherosclerotic ischemia/healthy	84/50	[74]
IL-37 transfection inhibits inflammation by increasing the expression of antioxidant enzyme (superoxide dismutase) and decreasing MDA in glucose-treated podocyte cells.	Podocyte cells		[75]
IL-37 inhibits NF-κB phosphorylation by downregulating the ox-LDL-activated TLR4-NF-κ B signaling pathway, reduces the expression of the osteogenic transcription factor RUNX2/ALP, and inhibits VSMC osteogenic transformation, thereby reducing the level of cellular calcification.	HA-SMC		[76]
IL-37 suppresses macrophage ferroptosis by improving oxidative stress. IL-37 inhibited HG/ox-LDL-induced ferroptosis in mouse bone marrow-derived macrophages. IL-37 promotes NRF2 activation via enhancing NRF2 nuclear translocation. IL-37 promotes NRF2 transfer from the cytoplasm to the nucleus. IL-37 inhibits ferroptosis of macrophages by activating the NRF2 pathway. IL-37 attenuates atherosclerosis progression in diabetic *ApoE*^−/−^ mice.	Male *ApoE*^−/−^ mice with and without diabetes and atherosclerosis, THP-1 cells		[77]

Abbreviations: MDA—malondialdehyde, RCAECs—rat Coronary Artery Endothelial Cells, ERS—endoplasmic reticulum stress, HA-CMC—human aortic smooth muscle cells, CAD—coronary artery disease, FRAP—ferric-reducing antioxidant power, Tregs—Regulatory T cells, HG—High glucose, NRF2—nuclear factor erythroid 2-related factor 2, NF-kB—nuclear factor kappa B.

### 3.8. IL-1Ra

IL-1Ra is a member of IL-1 family. It is stated to play a central role in inflammation relevant to atherosclerosis development, because the IL-1α and IL-1β (members of IL-1 family) response is tightly controlled by the expression of IL-1Ra [78]. IL-1Ra is produced by various cell types, including fibroblasts, monocytes, mast cells, neutrophils, B and T cells, macrophages, endothelial or epithelial cells [78,79]. Two forms of IL-1Ra have been described: secretory and intracellular [80]. The secretory form counteracts IL-1α/IL-1β–mediated pro-inflammatory signaling that drives immune cell recruitment, endothelial activation, and chronic inflammation in atherosclerotic plaques [78]. The mechanism of action of the other three intracellular forms is less clear.

IL-1Ra is thought to have a protective effect against the progression of atherosclerosis by reducing chronic inflammation in the artery wall and inhibiting the formation of fatty streaks in arteries too [79,81] (Table 7). The gene encoding IL-1Ra, called IL-1RN, is identified as a hub gene associated with atherosclerosis in human bioinformatics analyses. IL-1RN showed significantly higher expression in human atherosclerotic tissues and in foam cells compared with controls and is correlated with immune cell infiltration patterns relevant to atherogenesis [82].

Several review sources clearly describe the IL-1–IL-1Ra signaling mechanism in cells. They indicate that IL-1Ra inhibits IL-1β-induced nuclear factor kappa B (NF-kB) activation, reduces inflammasome signaling, and cytokine secretion in endothelial and monocyte/macrophage models. At the cellular level, IL-1Ra is recognized as a critical endogenous inhibitor of IL-1 mediator inflammatory signaling. By competitively binding to the IL-1 type I receptor without triggering further signal transduction, IL-1Ra prevents the activation of NF-κB and Mitogen-Activated protein kinase (MAPK) signaling pathways induced by IL-1α and IL-1β. In vascular endothelial cells and macrophages, these pathways regulate and promote the expression of adhesion molecules, cytokine secretion, oxidative stress, and foam cell formation all key processes in early atherogenesis or atherosclerotic plaque development (Table 7).

In vitro studies have shown that increased IL-1Ra availability inhibits proatherogenic cellular responses. Thus, there is a positive correlation between the inhibition of atherosclerotic plaque development and the onset or progression of the disease.

**Table 7:** Main findings from recent cell culture, animal and human studies about IL-1Ra.

Found Peculiarities of IL-1Ra	Cell Type	References
Competitive inhibition of IL-1 signaling reduces downstream NF-κB and MAPK pathway activation in vascular cells. IL-1Ra counteracts pro-inflammatory IL-1 effects, limiting cell surface adhesion molecule induction.	Endothelial cells/immune cells.	[83]
Elevated macrophage S1P_1_ signaling correlates with increased IL-1Ra production and an anti-atherogenic macrophage phenotype, reducing lesion size in LDL receptor–deficient mice.	Mice with S1P_1_ overexpression.	[84]
Higher IL-1Ra serum levels independently associate with total cholesterol, triglycerides, apolipoproteins B and C-III, and disease activity indicators.	Patients with rheumatoid arthritis n = 430.	[85]

Abbreviations: MAPK—Mitogen-Activated protein kinase, S1P_1_—Sphingosine-1-phosphate, LDL—Low-Density Lipoprotein.

In chronic inflammation mice models, such as a diet-induced atherosclerosis model, macrophages can switch to a more anti-inflammatory state, and this change is associated with higher levels of IL-1Ra. Increased IL-1Ra levels help block the harmful effects of IL-1, reduce inflammation, and activate processes that normally promote tissue damage. This reduces inflammation in blood vessels and slows the progression of atherosclerotic lesions [84]. This suggests that increasing IL-1Ra levels may help protect against inflammation-induced diseases by calming and suppressing the immune response and maintaining healthy blood vessels.

We managed to find only one study of IL-1Ra in humans. The research was a cross-sectional clinical study, involving 430 patients with rheumatoid arthritis (RA). Higher blood levels of IL-1 receptor antagonist (IL-1Ra) were associated with greater disease activity, reflecting more pronounced systemic inflammation. IL-1Ra concentrations also correlated with traditional CVD risk factors, including higher body mass index, abdominal obesity, elevated cholesterol, triglycerides, and apolipoproteins B and C-III. However, after adjusting for confounding factors, IL-1Ra levels were not significantly linked to atherosclerosis measures, such as carotid intima-media thickness or plaque presence. Higher IL-1Ra levels may reflect the interplay between chronic inflammatory activity and conditions related to it, and are associated with traditional CVD risk factors, but they are not directly linked to early signs of atherosclerosis [85].

In conclusion, IL-1Ra acts as a key endogenous inhibitor of IL-1–mediated inflammatory signaling by blocking NF-κB and MAPK pathways. This reduces cytokine secretion, oxidative stress, adhesion molecule expression, and foam cell formation in endothelial cells and macrophages, all of which reduces vascular inflammation and slows the progression of atherosclerotic lesions. Despite results on humans of atherosclerosis levels appear to mirror the degree of inflammation and are linked to common CVD risk factors such as obesity and dyslipidemia, however, IL-1Ra does not directly indicate early vascular changes, suggesting that while it reflects the inflammatory and metabolic burden, it may not serve as a direct marker of atherosclerosis. More detailed studies are needed to confirm these results in patients with atherosclerosis and without comorbidities.

### 3.9. IL-36Ra

IL-36 signal through the IL-36 receptor (IL-36R), a heterodimer formed of IL-1Rrp2 and an accessory coreceptor protein, IL-1RacP [86]. This receptor is also widely expressed at low levels in many organs and cell types, including leukocytes and vascular endothelial cells. Downstream intracellular signaling leads to NF-κB and MAPK activation and subsequent secretion of multiple potent proinflammatory mediators, including TNF-α, IL-1β, IL-6, and IL-8. The actions of the 3 agonists can be inhibited by a naturally occurring antagonist, namely the IL-36 receptor antagonist (IL-36Ra) [87]. They underpin the importance of careful endogenous management of the IL-36 pathway [87]. Both competitively bind the IL-1Rrp2 component of the heterodimer receptor, preventing recruitment of the accessory coreceptor IL-1RAcP and thus inhibiting subsequent intracellular signaling [87]. IL-36Ra can be secreted by dendritic, epithelial, endothelial cells, fibroblasts, keratinocytes, monocytes, and macrophages, and is therefore present in a wide range of tissues and has an important role in the regulation of inflammation and immune reactions [88,89]. Cell-based studies demonstrate that IL-36Ra acts as a natural inhibitor by binding to the IL-36 receptor and preventing the signaling of pro-inflammatory IL-36 cytokines (IL-36α, IL-36β and IL-36γ) without triggering downstream signaling, reducing inflammation and endothelial damage [78].

Studies in murine models of myocardial ischemia–reperfusion injury (IRI) and atherosclerosis demonstrate that IL-36Ra takes place in limiting vascular and cardiac injury. In these models, IL-36 and IL-36R were upregulated in cardiomyocytes and vascular endothelial cells following injury, with expression increasing with age and being more pronounced in female hearts, highlighting age- and sex-dependent amplification of pro-inflammatory signaling [87,90]. IL-36Ra mechanistically conferred vasculoprotection by limiting endothelial ROS damage and VCAM-1 expression was assessed in both adult and aged hearts of mice [87]. Pharmacological or genetic enhancement of IL-36Ra improves cardiovascular outcomes in murine models by limiting IL-36–driven inflammation. In myocardial ischemia–reperfusion injury and atherosclerosis models, IL-36Ra reduces vascular inflammation and tissue damage and promotes more stable vascular lesions [78]. IL-36Ra treatment led to a significant decrease in infarct size in both adult and aged mice. Since 1 dose of IL-36Ra was administered during the ischemic period, it is plausible that IL-36 could be targeted during percutaneous coronary intervention (PCI) procedures and be therapeutically efficacious in a clinical setting [91]. Data highlights a notable benefit to the coronary microcirculation and infarct size with early administration of IL-36Ra, which importantly is maintained in the presence of an aged comorbidity. This indicates that early intervention with an IL-36R inhibitor is worth considering for future clinical investigations [87]. The detailed mechanisms and experimental models are summarized in Table 8.

In humans, diabetes mellitus is characterized by chronic inflammation in the pancreas and adipose tissue, driven by excess glucose and free fatty acids. This pro-inflammatory milieu promotes the production of cytokines such as IL-1β, IL-6, TNF-α, and macrophage chemotactic protein-1, thereby contributing to insulin resistance and pancreatic β-cell apoptosis. As an anti-inflammatory member of the IL-36 family, IL-36Ra may counteract these processes by inhibiting IL-36–mediated signaling, ultimately reducing inflammation and potentially preserving β-cell function while limiting insulin resistance. Although these observations highlight the potential relevance of IL-36Ra in human metabolic and vascular disease, further studies are required to define its precise cellular targets and mechanisms in the context of cardiovascular complications of diabetes [95].

Evidence from human cardiovascular tissue analyses further supports the relevance of the IL-36/IL-36Ra axis. Analysis of left ventricular tissue from patients with advanced heart failure undergoing left ventricular assist device (LVAD) implantation demonstrated that components of the IL-36/IL-36Ra pathway are present and active in the human heart [94]. Although IL-36Ra was not directly modulated or administered in these human cardiac tissue samples, the detection of IL-36 signaling in diseased human myocardium supports the translational relevance of targeting this axis [94]. However, the lack of functional or interventional human data highlights the need for future studies to evaluate the specific role of IL-36Ra in cardiovascular disease progression and therapy.

In summary, cell models show IL-36Ra as a natural inhibitor of IL-36–mediated cardiovascular inflammation and support its potential as a therapeutic target for reducing endothelial dysfunction, microvascular injury, and inflammation. Human heart studies indicate that IL-36Ra may reduce inflammation in diabetes, while in failing hearts, IL-36/IL-36Ra components are present and show sex-related differences. However, functional effects of IL-36Ra in atherosclerosis in humans must be determined. Future studies should clarify how IL-36Ra interacts with sex-specific pathways and explore IL-36Ra as biomarker and IL-36 as therapeutic target through precise delivery strategies for personalized therapy.

### 3.10. IL-38

**Figure 4:**
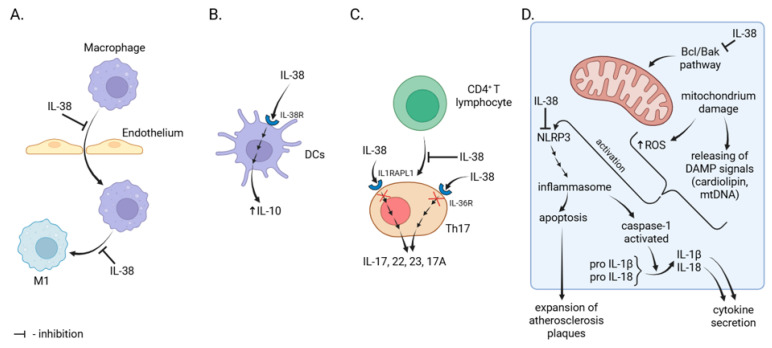
Effect of IL-38 against atherosclerosis. Abbreviations: M 1—macrophage with pro-inflammatory phenotype, DCs—dendritic cells, IL1RAPL1—Interleukin-1 Receptor Accessory Protein-Like 1, NLRP3—NOD-like receptor family, containing pyrin domain, ROS—reactive oxygen species, DAMP—Damage-Associated Molecular Patterns, mtDNA—Mitochondrial deoxyribonucleic acid, the blue background in part D represents the interior of the cell. Created using BioRender.

**Figure 5:**
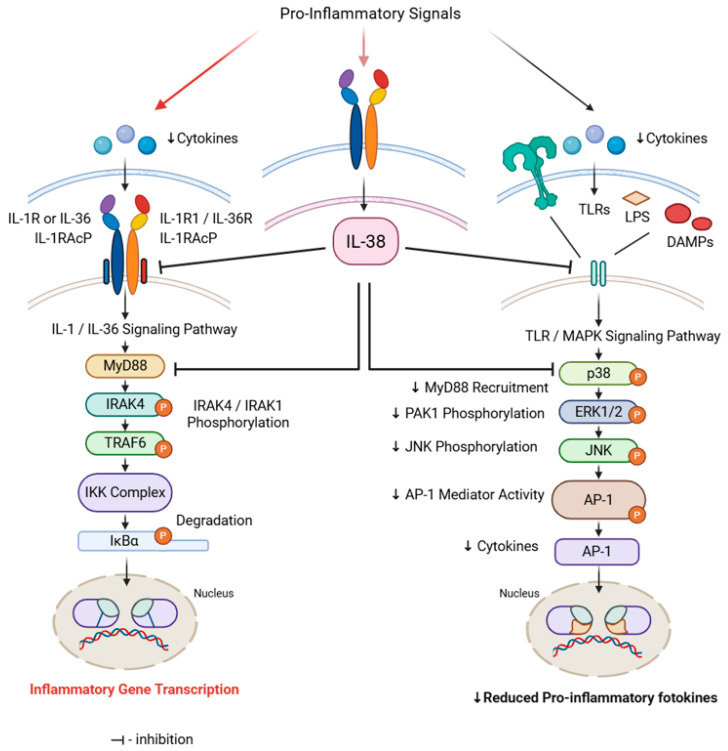
IL-38 inhibits pro-inflammatory signaling pathways. Abbreviations: IL-38.—Interleukin-38, MAPK—Mitogen-Activated Protein Kinase, p38—p38 Mitogen-Activated Protein Kinase (MAPK14), ERK1/2—Extracellular Signal-Regulated Kinase 1/2, JNK—c-Jun N-terminal Kinase, AP-1—Activator Protein-1, IL-1—Interleukin-1, IL-36—Interleukin-36, IL-1R1—Interleukin-1 Receptor Type 1, IL-36R—Interleukin-36 Receptor (IL1RL2), IL-1RAcP—Interleukin-1 Receptor Accessory Protein, MyD88—Myeloid Differentiation Primary Response Protein 88, IRAK4—Interleukin-1 Receptor-Associated Kinase 4, IRAK1—Interleukin-1 Receptor-Associated Kinase 1, TRAF6—TNF Receptor-Associated Factor 6, IKK Complex—IκB Kinase Complex, IκBα—Inhibitor of Nuclear Factor Kappa-B Alpha, TLRs—Toll-Like Receptors, LPS—Lipopolysaccharide, DAMPs—Damage-Associated Molecular Patterns, IL-1β—Interleukin-1 Beta. Created using BioRender.

IL-38 is the last member of anti-inflammatory ILs group. It seems its’ role in atherosclerosis is revealed broader and is well-reviewed by Xiao-Hong Zhang with coauthors [96]. In brief, authors have shown the structure and function of IL-38 in detail and systematically reviewed the mechanism and effect of IL-38 against atherosclerosis revealing the potential applicability of IL-38 in clinical treatment. Authors reviewed that IL-38 may bind three receptors: IL-1R1, IL-36R, and IL1RAPL1 and act as receptor antagonist and blocks signal transduction. IL-38 is secreted by B cells, macrophages, and cardiomyocytes in response to changes in the atherosclerotic microenvironment. Some fields of IL-38 action were pointed: (1) IL-38 can reduce macrophage infiltration under the endothelium, inhibit their transition to an inflammatory phenotype and inhibit the activation of the NLRP3 (NOD-like receptor family, containing pyrin domain what can be found in macrophages, monocytes, neutrophils, endotheliocytes and is important for inflammasome formation and inflammation activation, Figure 4A); (2) upon stimulation with IL-38, the maturation of DCs is reduced and the expression of the anti-inflammatory cytokine IL-10 is increased (Figure 4B); (3) IL-38 reduces the differentiation of CD4+ T lymphocytes into Th17 cells and antagonizes IL-36R and IL1RAPL1 (Interleukin-1 Receptor Accessory Protein-Like 1) to inhibit the production of inflammatory cytokines, such as IL-17, IL-22, IL-23, and IL-17A (Figure 4C); (4) by regulating the Bcl/Bax pathway, IL-38 reduces apoptosis to inhibit the expansion of atherosclerotic plaques (Figure 4D); (5) IL-38 limits the size and number of adipocytes and reduces the accumulation of lipids in atherosclerotic lesions to ameliorate atherosclerosis, which implies that it can limit atherosclerosis through multiple pathways. Accordingly, recombinant IL-38 (aa 5–152) was shown to have a powerful anti-atherosclerotic effect.

It was revealed these signaling pathways of IL-38 latter.

1. IL-36R/competitive antagonism—blocks the formation of the IL-36R/IL-1RAcP complex. IL-38 can bind to IL-36R (and common co-receptors in the complex), thereby preventing the signaling of pro-inflammatory IL-36 cytokines. This prevents the formation of an active receptor complex, which normally requires IL-1RAcP (IL-1 receptor accessory protein) [91].
2. Normally, IL-1/IL-36/IL-1R1 signaling binds to Myeloid Differentiation Factor 88 (MyD88). Emerging IL-1 receptor-associated kinase ¼ (IRAK1/4) is activated, the signal is transmitted via tumor necrosis factor receptor associated factor 6 (TRAF6) to the IκB kinase (IKK) complex, IκBα phosphorylation/degeneration and NF-κB (p65) translocation to the nucleus occur transcription of inflammatory genes. Studies of IL-38 show that IL-38 reduces the activation of this pathway (less IRAK/TRAF6 phosphorylation, less NF-κB nucleation/activation, Figure 5) [97].
3. MAPK cascades (p38, ERK1/2, JNK)—inhibition. IL-1 family signaling also activates MAPK pathways (p38, ERK, JNK), which promote cytokine synthesis via AP-1 interactions. Several studies report that IL-38 reduces p38/ERK/JNK phosphorylation, which decreases AP-1 mediator activity and inflammatory cytokine production (Figure 5) [98].
4. NOD-like receptor (NLR) and pyrin domain-containing protein 3 (NLRP3) inflammasome and IL-1β maturation—inhibition. In some systems, IL-38 reduces NLRP3 inflammasome activation, decreases caspase-1 activity, and consequently reduces the release of mature IL-1β—this is another way in which IL-38 inhibits IL-1-mediated inflammation (the mechanism may be either direct or indirect via reduced upstream signals NF-κB/MAPK, Figure 4D) [98].
5. Interaction with IL1RAPL1 (TIGIRR-2) and additional inhibitory signals. Some studies suggest that IL-38 may form complexes with IL1RAPL1 (also known as TIGIRR-2) together with IL-36R, which may initiate additional inhibitory or modulatory signaling pathways that influence the immune response (Figure 4C) [99].
6. Effect on immune cell reduction in Th17, alteration in macrophage polarization. Functional consequences: IL-38 often reduces the Th17 response and promotes a tolerogenic/M2-like macrophage phenotype—this is a partial consequence of previous suppressions at the NF-κB/MAPK/NLRP3 level (Figure 4A) [100].

IL-38 was shown can inhibit preadipocyte differentiation by inducing GATA motif-binding transcription factor 3 (GATA-3) expression, reducing triglyceride synthesis and adipocyte size to reduce obesity and insulin resistance. It is well known that obesity and diabetes are major risk factors for CVD. Moreover, IL-38 can reduce the release of IL-1β, IL-6 and monocyte chemoattractant protein-1 (MCP-1), as well as the levels of total cholesterol, triacylglycerol, and LDL, thereby improving lipid and glucose metabolism and reducing the risk of CVD [90,101,102].

Although the specific role of IL-38 in angiogenesis is still under investigation, some studies suggest that IL-38 may reduce endothelial damage by inhibiting endothelial cell proliferation and migration and by reducing the release of angiogenic factors. Studies suggest that IL-38 may exert anti-inflammatory effects by reducing the expression of IL-8 and TNF-α. These cytokines promote angiogenesis by stimulating endothelial cell proliferation, migration and new blood vessel formation [90]. Thus, IL-38 may limit the activation of angiogenesis. In addition, IL-38 may inhibit NLRP3 inflammasome activation, thereby suppressing macrophage-mediated inflammation and reducing cardiomyocyte apoptosis [102]. The most important recent findings in cells, mice and humans are presented in Table 9.

Overall, across all the studies, IL-38 consistently suppresses pro-inflammatory signaling, limits oxidative stress, and reduces macrophage-driven vascular damage. IL-38 appears to act through IL-1 family–related receptors and regulates multiple intracellular pathways, including PPAR-γ/NRF2-mediated antioxidant signaling [104], SIRT6/HO-1 [105] anti-inflammatory pathways, and regulation of macrophage polarization. Despite growing interest in the possible involvement of IL-38 in atherosclerosis and its potential as a therapeutic target, large scale research is needed to determine its precise role, underlying receptor biology, tissue-specific effects, therapeutic relevance and long-term outcomes in humans only with atherosclerosis.

## 4. Integrating Insights

Detailed knowledge of anti-inflammatory ILs actions on cells, tissue specificity, receptor usage, downstream signaling and what still needs to be clarified is presented in Table 10. The following subsections address the mechanisms of action of anti-inflammatory ILs at both the molecular and organismal levels and evaluate their potential as diagnostic biomarkers and therapeutic targets in atherosclerosis.

### 4.1. Protective Mediators or Compensatory Markers?

Anti-inflammatory ILs are produced by a variety of cell types. In turn, these ILs can act on multiple target cells and exert anti-inflammatory effects via intracellular signaling pathways. Consequently, it is often challenging to distinguish which ILs are secreted as part of a compensatory response, and which have a causative protective role in atherosclerosis. Human observational data largely supports the concept of compensatory elevation rather than causative protection of IL-5, IL-10, IL-19. Circulating IL-5 levels show weak or absent associations with incident cardiovascular events, suggesting that increased IL-5 reflects secondary immune activation rather than a primary protective driver [23]. However, presence of plaque at the carotid bifurcation was associated with lower IL-5 and IL-5 deficiency resulted in increased plaque development at sites of oscillatory blood flow in *Apoe*^−/−^ mice suggesting a protective role for IL-5 in plaque development [23]. This interpretation is reinforced by the broader context of type-2 immunity, where IL-5 is produced downstream of inflammatory cues (e.g., ILC2 activation), indicating that its elevation may be context-dependent and reactive rather than mechanistically decisive [116]. Circulating IL-10 is produced in response to systemic inflammation and correlates weakly with other inflammatory mediators, suggesting that its elevation is reactive and context-dependent too [117]. Nevertheless, some observational settings show inverse associations between IL-10 and plaque burden, supporting a protective association without establishing causation [40]. These findings support the interpretation of IL-10 as a compensatory immunoregulatory signal in human atherosclerosis. In contrast, experimental studies consistently demonstrate causative atheroprotection of IL-5 and IL-10. Moreover, disruption of IL-5 signaling or its upstream ILC2 axis leads to impaired IgM responses and increased atherosclerosis, supporting a direct mechanistic role in vascular protection [24]. IL-10 suppresses macrophage activation, pro-inflammatory cytokine production, and matrix-degrading enzymes, thereby reducing lesion formation and promoting plaque stabilization [36]. Recent studies demonstrate that targeted overexpression of IL-10 in macrophages or plaque-directed delivery systems reduces plaque size, inflammation, and necrotic core formation, directly confirming its mechanistic role [32]. However, emerging data indicate context-dependent effects: IL-10 may also reduce collagen synthesis and thereby impair plaque stability under certain conditions, highlighting a dual and stage-dependent role [118].

Interleukin-13 (IL-13) exhibits a context-dependent role in atherosclerosis, with evidence supporting both causative protection and complex, potentially adverse vascular effects. Experimental studies consistently indicate causative atheroprotection. IL-13 promotes alternative (M2) macrophage polarization, enhances oxidized LDL clearance, reduces vascular cell adhesion molecule-1 (VCAM-1)–mediated monocyte recruitment, and increases plaque collagen content, thereby contributing to a more stable plaque phenotype. Moreover, IL-13 deficiency accelerates atherosclerosis in murine models, supporting a direct protective role independent of lipid levels [43]. In humans, however, data is largely associative and suggest compensatory regulation. Lower circulating IL-13 levels are associated with increased carotid intima-media thickness, implying a protective correlation but not causation [46]. This supports the interpretation that IL-13 may function as a marker of anti-inflammatory capacity, rather than a primary determinant of disease progression. Mechanistically, IL-13 acts through the JAK/STAT6 pathway, modulating endothelial function, smooth muscle cell behavior, and macrophage phenotype. However, emerging evidence indicates dual effects, as IL-13 may also contribute to endothelial dysfunction, oxidative stress, and vascular remodeling under certain conditions [119]. As a biomarker, IL-13 has limited clinical utility due to variability and lack of validation in large cohorts. As a therapeutic target, its role remains uncertain: while enhancing IL-13 signaling may promote atheroprotection, clinical inhibition of IL-13 (e.g., in allergic diseases) has uncleared cardiovascular consequences [119].

From a clinical and observational perspective, increased IL-19 levels may represent a compensatory anti-inflammatory response to ongoing vascular injury rather than a primary driver of protection. Consistent with this, IL-19 is often upregulated alongside inflammatory pathways and immune activation, indicating a role as a reactive immunomodulatory cytokine rather than a direct determinant of disease progression [120]. IL-19 expression appears to be inducible in inflammatory environments, particularly within vascular cells and macrophages exposed to pro-atherogenic stimuli. This suggests that increased IL-19 levels may represent a compensatory anti-inflammatory response to ongoing vascular injury rather than a primary driver of protection. IL-19 has been shown to inhibit vascular smooth muscle cell (VSMC) proliferation and migration, reduce pro-inflammatory cytokine production, and modulate macrophage responses toward a less inflammatory phenotype. These effects collectively attenuate plaque formation and progression, indicating a direct mechanistic role in limiting atherogenesis [121]. IL-19 has not been clinically validated as a cardiovascular biomarker and is primarily characterized in experimental settings, with expression that is highly context-dependent.

Circulating IL-37 levels are increased in patients with atherosclerosis and correlate with disease severity, indicating association but not necessarily causation [74]. Similarly, IL-37 is induced by inflammatory stimuli and elevated in acute coronary syndromes, supporting its role as a reactive anti-inflammatory mediator [122]. IL-38 shows comparable behavior as an inflammation-responsive cytokine that modulates key signaling pathways (e.g., NF-κB, MAPK), suggesting context-dependent expression rather than disease specificity [96]. In contrast, clinical data on IL-35 remain limited, and its circulating dynamics in human atherosclerosis are less defined, highlighting a major knowledge gap. IL-35 is implicated in immune resolution pathways and anti-inflammatory networks linked to regulatory immunity, suggesting a potential causative protective role that requires further direct validation [123].

Experimental studies support causative atheroprotection for IL35, 37 and 38 cytokines, albeit with different levels of evidence. IL-37 has the strongest mechanistic support: recombinant or transgenic IL-37 reduces plaque size, suppresses macrophage infiltration, and promotes regulatory T-cell responses in murine models [124]. IL-38 similarly attenuates atherosclerosis by inhibiting macrophage M1 polarization, improving cholesterol efflux, and reducing plaque burden [103].

IL-1Ra and IL-36Ra are endogenous inhibitors within the IL-1 cytokine family, illustrating both compensatory elevation and causative protection, but with markedly different levels of evidence. Clinically, IL-1Ra is elevated in cardiometabolic states and correlates with inflammatory burden, indicating compensatory upregulation in response to IL-1–driven inflammation rather than direct causality [125]. Such associations, including links with coronary heart disease risk, support association without clear protective causation. In contrast, human data for IL-36Ra in atherosclerosis are scarce, representing a major knowledge gap [78]. Experimental evidence supports causative protection, particularly for IL-1 pathway inhibition: blocking IL-1 signaling reduces vascular inflammation and atherosclerotic progression [126]. IL-36Ra exerts anti-inflammatory effects by competitively inhibiting IL-36 signaling and downstream NF-κB/MAPK activation [78], though direct in vivo atherosclerosis data remains limited.

Thus, the apparent paradox can be reconciled: IL-5, IL-10, IL-19 are causative in controlled experimental systems, but in humans often appears as a compensatory correlate of immune activation. IL-13 appears mechanistically protective but clinically ambiguous, with unresolved questions regarding its net vascular effects, stage-specific actions, and translational potential. IL-35, IL-37, and IL-38 share a common pattern: upregulated expression likely reflects compensatory immune regulation, while experimental data support causative atheroprotection. However, their biomarker utility is limited, and their therapeutic promise remains largely preclinical, emphasizing the need for well-designed human studies to clarify their translational relevance. Both IL-1Ra and IL-36Ra compensatory elevation and causative protection, but with markedly different levels of evidence. As biomarkers, IL-1Ra and IL-36Ra lack specificity due to inflammation-dependent expression. Therapeutically, IL-1Ra is clinically translated (e.g., anakinra), whereas IL-36Ra remains preclinical, highlighting unresolved translational potential.

### 4.2. Shared and Divergent Anti-Inflammatory Mechanisms of ILs in Atherosclerosis

Atherosclerosis is a chronic inflammatory disease driven by complex cytokine networks integrating JAK/STAT, NF-κB, MAPK, and inflammasome pathways [127]. Within this context, anti-inflammatory IL exhibits both overlapping and distinct signaling mechanisms.

Shared mechanisms include modulation of macrophage polarization and inhibition of pro-inflammatory signaling. IL-10, IL-35, IL-37, and IL-38 broadly suppress NF-κB and MAPK pathways in macrophages, promoting an M2-like phenotype and reducing cytokine-mediated vascular inflammation [36,116]. A central overlap exists among IL-10 family and Th2-associated cytokines (IL-5, IL-10, IL-13, IL-19, IL-35), which predominantly modulate atherosclerosis through JAK/STAT-dependent signaling. IL-10 signals mainly via JAK1/STAT3 and suppresses NF-κB–driven inflammatory gene expression in macrophages, endothelial cells, and vascular smooth muscle cells, thereby reducing plaque formation and immune cell infiltration [119]. IL-5 further overlaps functionally by promoting IgM production against oxidized LDL, limiting foam cell formation and plaque progression [121]. IL-19, as part of the IL-10 cytokine family, signals via IL-20 receptor complexes and is linked to STAT3-mediated anti-inflammatory responses, contributing to vascular homeostasis and reduced inflammatory activation in atherosclerotic lesions [128]. IL-35 exerts anti-atherogenic effects by inhibiting MAPK signaling and reducing VCAM-1 expression in endothelial cells, thereby limiting leukocyte recruitment and vascular inflammation [121]. Thus, these cytokines converge on suppressing endothelial activation and immune cell recruitment, though they utilize partially distinct downstream mediators (STAT3, STAT6, MAPK inhibition).

In contrast, IL-1 family members (IL-1Ra, IL-36Ra, IL-37, IL-38) primarily regulate atherosclerosis through inhibition of innate immune signaling, particularly NF-κB and inflammasome pathways. IL-1Ra and IL-36Ra act as receptor antagonists that block IL-1/IL-36 signaling, thereby preventing activation of pro-inflammatory cascades central to plaque development [78]. These mechanisms directly counteract IL-1β-driven inflammation, a key driver of atherosclerosis progression [129]. IL-1Ra and IL-36Ra competitively inhibit IL-1 and IL-36 receptor signaling, preventing leukocyte recruitment and vascular injury. IL-5, IL-13, and IL-19 act primarily within type-2 immunity, promoting eosinophil-mediated anti-inflammatory effects and supporting regulatory T-cell function [23,116]. Additionally, IL-37 and IL-38 extend this anti-inflammatory paradigm via broader intracellular and extracellular signaling modulation. IL-37 suppresses atherosclerosis progression by activating NRF2-dependent antioxidant pathways and inhibiting macrophage ferroptosis, while simultaneously reducing IL-1β and IL-18 production [77]. This highlights a distinct mechanism linking cytokine signaling to oxidative stress regulation. IL-38, similar to IL-36Ra, is thought to inhibit IL-36 receptor signaling and downstream Th17-related cytokine production, thereby attenuating vascular inflammation (as summarized in recent IL-1 family reviews) [78].

**Figure 6:**
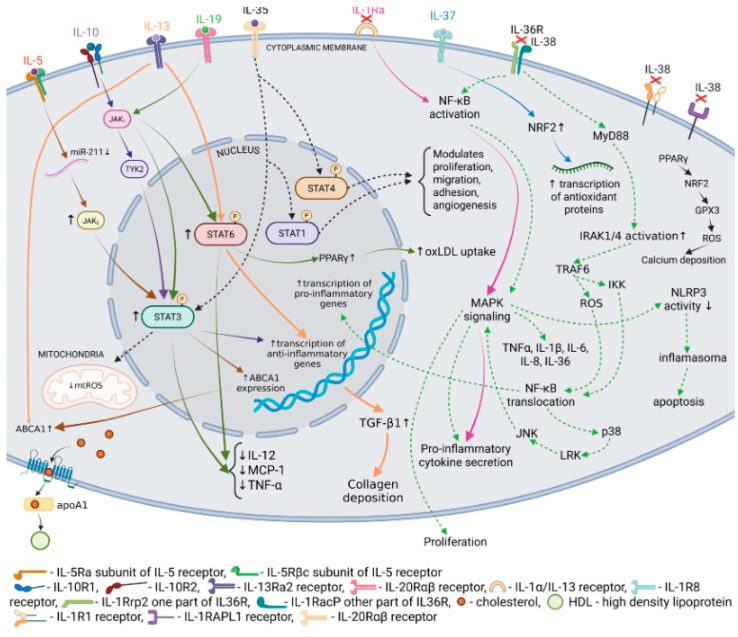
Shared and Divergent Anti-Inflammatory pathways of ILs in Atherosclerosis. IL-5 reduces the level of miR-211 to increase JAK2 expression and activates STAT3 in macrophages, which consequently increases ABCA1 expression and promotes cellular cholesterol efflux to apoA-1 and—high density lipoprotein (HDL). Cholesterol decreasing in macrophages reduce macrophage-mediated inflammation in atherosclerotic plaques by increasing ABCA1 expression. Transcription of anti-inflammatory genes (IL-10 action) and suppression proinflammatory cytokines. chemokines and chemokine receptors IL-13 signaling via the IL-13Ra2 has been shown to induce TGF-b1 production in macrophages via a STAT6-independent pathway, leading to collagen deposition. Accordingly, IL-10 encourages ABCA1 action. IL-19-mediated increase in scavenger receptor expression and cholesterol uptake in macrophages (BMDMs) are driven by PPARƴ activation (through the STAT6). IL-19 can increase oxLDL uptake in macrophages (because of PPARƴ activation). IL-19-mediated increase in STAT3 expression leads to decreasing of IL-12, MCP-1 and TNF-α synthesis. IL-35 blocks LPC-induced mitochondrial reactive oxygen species (mtROS), which are required for the induction of site-specific histone 3 lysine 14 (H3K14) acetylation, increased binding of pro-inflammatory transcription factor AP-1 in the promoter of ICAM-1, and induction of ICAM-1 transcription in. IL-1Ra prevents the activation of NF-κB and MAPK signaling pathways induced by IL-1α and IL-1β. In vascular endothelial cells and macrophages, these pathways regulate and promote the expression of adhesion molecules, cytokine secretion, oxidative stress, and foam cell formation all key processes in early atherogenesis or atherosclerotic plaque development. IL-36R signaling leads to NF-κB and MAPK activation and subsequent secretion of multiple potent proinflammatory mediators, including TNF-α, IL-1β, IL-6, and IL-8 and pro-inflammatory IL-36 cytokines (IL-36α, IL-36β and IL-36γ). IL-38 appears to act through IL-1 family–related receptors and regulates multiple intracellular pathways, including PPAR-γ/NRF2-mediated antioxidant signaling. NLRP3, NF-kB/MAPK and MyD88-NF-kB pathways take part in IL-38 signaling. The specific receptor mediating IL-38 activity in the PPARγ → NRF2 → GPX3 → ROS → calcification pathway has yet to be identified. Abbreviations: PPAR—peroxisome proliferator-activated receptor; BMDMs—bone marrow-derived macrophages; MCP-1—Monocyte chemoattractant protein-1; TNF-α—Tumor necrosis factor α; LPCs—Lysophosphatidylcholines; ICAM-1—intercellular adhesion molecule-1; NF-κB—Nuclear factor-κB; MAPK—mitogen-activated protein kinase; GPX3—Glutathione Peroxidase 3; PPAR-γ—Peroxisome Proliferator-Activated Receptor γ; NRF2—Nuclear factor erythroid 2-related factor 2; ROS—Reactive Oxygen Species; ABCA1—ATP binding cassette transporter A1, TYK2—tyrosine kinase 2, TGF-β1—tumor necrosis growth factor β1. Created using BioRender.

Divergent mechanisms reflect cytokine-specific pathways and cellular targets. IL-5 preferentially affects B1 cells and IgM production, limiting oxidized LDL deposition [23], whereas IL-13 influences smooth muscle cell proliferation and extracellular matrix remodeling [116]. IL-37 and IL-38 act as broad immunomodulators with systemic effects, whereas IL-36Ra and IL-1Ra are receptor-specific antagonists with more targeted anti-inflammatory roles. IL-19 exhibits both type-2 immunoregulatory and endothelial-protective properties, uniquely bridging immune modulation and vascular homeostasis [116]. Overall, these ILs demonstrate overlapping anti-inflammatory actions on immune cells but diverge in tissue specificity, receptor usage, and downstream signaling, emphasizing the importance of pathway-targeted approaches in therapeutic development (Figure 6). Translational challenges remain, including cells’ species differences, compensatory cytokine networks, and limited human validation [130].

Overall, two major signaling clusters emerge in atherosclerosis:

(1) JAK/STAT-centered immunoregulatory cytokines (IL-5, IL-10, IL-13, IL-19, IL-35), which primarily modulate adaptive immunity, macrophage polarization, and endothelial activation; and.

(2) IL-1 family inhibitory cytokines (IL-1Ra, IL-36Ra, IL-37, IL-38), which suppress innate immune signaling via receptor antagonism, NF-κB inhibition, and alternative pathways such as NRF2. Despite mechanistic differences, all these cytokines converge functionally on reducing vascular inflammation, limiting immune cell infiltration, and stabilizing atherosclerotic plaques, underscoring the redundancy and therapeutic potential of anti-inflammatory signaling networks in atherosclerosis [129].

### 4.3. Biomarker Relevance and Therapeutic Potential

The practical relevance of presented knowledge lies in its’ application to the study of anti-inflammatory ILs, both as circulating biomarkers of atherosclerosis progression and as potential therapeutic targets. Clinically, IL-5 and IL-10 perform poorly as a biomarker due to low predictive value and limited specificity for cardiovascular outcomes [23,117]. IL-5 levels are influenced by multiple type-2 immune processes (e.g., allergy), further reducing its utility in risk stratification [23]. IL-10 levels are influenced by diverse inflammatory states, limiting their utility for cardiovascular risk stratification [117]. Regarding biomarker relevance, none of IL-35, 37 and 38 are currently validated as robust cardiovascular biomarkers too. Their expression is highly context-dependent and influenced by systemic inflammation, limiting specificity. For instance, IL-37 elevation occurs across multiple inflammatory diseases, weakening its predictive value for atherosclerosis despite disease correlations [122]. IL-38 and IL-35 face similar limitations due to insufficient longitudinal and outcome-based human data, underscoring their status as associative rather than clinically actionable markers. As biomarkers, both IL-1Ra and IL-36Ra lack specificity due to inflammation-dependent expression.

In opposite, IL-5 retains significant therapeutic plausibility. Mechanistic studies highlight its central role within the IL-33/IL-25/TSLP–ILC2–IL-5–IgM axis, which promotes anti-inflammatory macrophage polarization and atheroprotective humoral immunity [116]. However, translation into cardiovascular therapy remains uncertain, as IL-5 is also a key mediator of eosinophilic inflammation and is already targeted in non-cardiovascular diseases, raising concerns about systemic modulation [131]. In contrast, IL-10 remains a promising therapeutic candidate, supported by strong anti-inflammatory and immunomodulatory effects. Advances in targeted delivery (e.g., LDL-bound IL-10 constructs or macrophage-based therapies) have demonstrated improved efficacy compared with systemic administration, which is limited by short half-life and pleiotropy [35]. By modulating macrophage polarization, inhibiting VSMC activation, and suppressing inflammatory signaling, IL-19 represents a plausible target for immunomodulatory therapy in atherosclerosis. However, as with other IL-10 family cytokines, its pleiotropic effects and context-dependent signaling raise challenges for clinical translation, including potential off-target immune modulation.

Conversely, IL-35, 37 and 38 ILs demonstrate therapeutic potential in experimental models. IL-37 and IL-38 directly suppress inflammatory signaling pathways and modulate macrophage phenotype, resulting in reduced plaque development [103]. IL-35, through its role in regulatory immune responses, may enhance anti-inflammatory pathways and plaque stability, although translational evidence remains sparse. Importantly, for IL-35, IL-37 and IL-38 cytokines, uncertainties persist regarding optimal delivery, systemic effects, and context-dependent actions, as well as discrepancies between human and animal data [122]. Therapeutically, IL-1Ra is clinically translated (e.g., anakinra), whereas IL-36Ra remains preclinical, highlighting unresolved translational potential.

### 4.4. Translational Limitations of Preclinical IL Modulation

A major challenge in translating cytokine-targeted strategies from preclinical models to clinical atherosclerosis lies in the discrepancy between robust mechanistic effects in animals and limited or inconsistent human outcomes. Across anti-inflammatory ILs, several recurring limitations emerge. First, context-dependent and compensatory expression complicates causal interpretation. IL-10 and IL-1Ra are upregulated in response to systemic inflammation, reflecting reactive immune regulation rather than primary protection [36,126]. Similarly, IL-5, IL-13, and IL-19 are induced within type-2 immune responses, often downstream of broader inflammatory networks, limiting their specificity as therapeutic targets [23,116]. For newer ILs (IL-35, IL-37, IL-38, IL-36Ra), human data remain sparse, and their circulating levels are influenced by multiple inflammatory conditions, further confounding interpretation. Second, species differences and model limitations significantly hinder translation. Murine atherosclerosis models (e.g., *ApoE*^−/−^, LDLR^−/−^) often exaggerate immune-driven mechanisms and do not fully recapitulate human plaque biology, particularly plaque rupture and chronic immune adaptation [130]. IL-37, which lack direct murine homologs, requires transgenic expression systems, limiting physiological relevance. Consequently, protective effects observed for IL-5, IL-10, IL-37, or IL-38 in mice may not translate directly to humans. Third, pleiotropy and pathway redundancy reduce therapeutic precision. These ILs act within overlapping cytokine networks (e.g., IL-1, IL-10, and type-2 immunity axes), meaning that modulation of a single cytokine often produces compensatory pathway activation. For example, IL-10 and IL-35 broadly suppress immune responses, raising concerns about immunosuppression, while IL-13 and IL-5 are linked to allergic pathways, complicating systemic targeting [116]. Fourth, biomarker limitations weaken clinical translation. Most of these cytokines lack specificity and predictive value for cardiovascular outcomes. IL-5, IL-10, and IL-1Ra show inconsistent associations with disease progression, while IL-19, IL-35, IL-37, IL-38, and IL-36Ra remain insufficiently validated in large human cohorts [23]. This limits their utility for patient stratification in therapeutic trials. Finally, delivery, dosing, and safety challenges remain unresolved. Additionally, IL-10 exhibits short half-life and systemic effects, necessitating targeted delivery strategies, while IL-1 pathway inhibition (e.g., anakinra, canakinumab) demonstrates that clinical efficacy is achievable but accompanied by infection risk and high cost [130].

## 5. Conclusions

The literature presents not only consistent findings regarding specific ILs when their effects are examined across different patient groups, including obesity, acute myocardial infarction, stabile angina pectoris, metabolic syndrome, and other conditions. In this article, we provide a comprehensive review of studies focusing exclusively on the role of anti-inflammatory ILs in the context of atherosclerosis. Taken together, findings from recent studies suggest that all the anti-inflammatory ILs: (1) take part in the regulation of cholesterol transport or oxLDL phagocytosis (IL-1Ra and IL-36Ra—indirectly); (2) affects different blood cells’ participation in the inflammation; (3) takes place in the remodelation of the arterial wall. These are the main pathways through which ILs exerts its anti-inflammatory effects. It seems that ILs action results in reduction of the inflammation (involving macrophage polarization into anti-inflammatory phenotype), correction of lipid metabolism, foam cell formation and limiting of endothelium dysfunction. The influence of each interleukin on the development of atherosclerosis depends on the extent of involvement of the cells secreting it in the atherosclerotic process, as well as on the inducers activating interleukin secretion. It appears that the intensity of atherosclerosis development depends on the combined effect of active factors, including the cells involved in this process and external factors acting as risk factors. The effect of a particular interleukin may also depend on the receptor through which it signals, and the cell types involved. We have shown the function of anti-inflammatory ILs in detail and systematically reviewed the shared and divergent action mechanism and effect against atherosclerosis with the hope of revealing the potential applicability of anti-inflammatory ILs in clinical diagnostics and treatment. The most promising for drug targeting of signaling pathways could be Il-35, IL-37 and IL-38. These ILs demonstrate therapeutic potential in experimental models. All the anti-inflammatory ILs seems could not be as diagnostic biomarkers for atherosclerosis yet. All of them are influenced by diverse inflammatory states, limiting its utility for atherosclerosis stratification. Additionally, IL-5, IL-10, and IL-1Ra exhibit inconsistent correlations with disease progression, whereas IL-19, IL-35, IL-37, IL-38, and IL-36Ra remain insufficiently validated in large human populations. As a result, their value for patient stratification in therapeutic trials is constrained. Furthermore, key challenges related to delivery, dosing, and safety remain unresolved.

## Figures and Tables

**Table 1:** Influence of IL-5 on atherosclerosis development.

Found Perculiarities of IL-5	n (in Humans)	References
IL-5 overexpression reduces the levels of IL-1, IL-6, IL-18, and TNF-a in humans. Was verified decreasing IL-5 expression in the aorta of patients with AAD.	Aortic tissue samples from normal donors (11) and patients with AAD (18).	[22]
There are no associations between levels of IL-5 and risk for development of coronary events or stroke during a 15.7 ± 6.3 years follow-up of randomly sampled person from the Malmö Diet and Cancer study. However, presence of a plaque at the carotid bifurcation was associated with lower IL-5 and IL-5 deficiency resulted in increased plaque development at sites of oscillatory blood flow in *ApoE*^−/−^ mice suggesting a protective role for IL-5 in plaque development. Significantly lower plasma IL-5 was in patients with carotid atherosclerotic plaque. Any association between IL-5 plasma levels and IgM against oxidation-specific epitopes. a weak significant association between IL-5 released from activated leukocytes and IgM against a malondialdehyde modified sequence of apo B-100. IL-5 per se can modulate endothelial cell phenotype and activity.	696 subjects randomly sampled from the Malmö Diet and Cancer study. Mice	[23,24]
Plasma IL-5 significantly inversely correlated with changes in carotid intima-media thickness.	High-risk individuals (n = 3534) free of clinically overt CVD at enrollment, in whom composite and segment-specific measures of cIMT were recorded at baseline and after 15 and 30 months.	[25]
Plasma IL-5 levels were significantly associated with antibody titers to copper oxLDL and IgM to malondialdehyde-modified LDL. Plasma IL-5 levels were found to be inversely associated with bifurcational IMT, and even after adjustments for traditional risk factors of atherosclerosis (age, gender, smoking, SBP, LDL, and body mass index), IL-5 remained an independent determinant of the mean bifurcational IMT (*p* = 0.010).	1011 people of OPERA study (86 subjects had CAD, 93 subjects were diabetic, the diseases of the other persons are not presented).	[26]

Abbreviations: AAD—acute aortic disecation, CAD—coronary artery disease, CVD—cardiovascular disease, cIMT—carotid intima-media thickness, SBP—systolic blood pressure, LDL—low density lipoprotein, oxLDL—oxidised low-density lipoprotein.

**Table 2:** Influence of IL-10 on atherosclerosis development.

Found Peculiarities of IL-10	Cell Type/Mice/Humans	References
Inhibits macrophage activation and reduces pro-inflammatory mediators such as TNF-α, IL-1β, IL-6, IL-8, MMPs, and foam cell formation. In addition, Reduces cholesterol accumulation in the vascular wall.	Macrophages (mouse atherosclerosis models)	[36]
Reduce inflammation, apoptosis and stimulates both the uptake (by up-regulating scavenger receptors) and efflux of cholesterol (by activating the PPARγ-LXR-ABCA1/ABCG1 pathway).	Primary human macrophages derived from monocytes	[37]
IL-10 stabilizes atherosclerotic plaques by protecting foam cells from apoptosis, enhancing lipid retention, and upregulating anti-apoptotic genes (Bfl-1, Mcl-1), which supports mitochondrial function.	Human macrophages derived from THP-1 cells and from ACS patient monocytes	[38]
Suppresses atherosclerotic plaque formation and reduces lipid accumulation in lesions by regulating cholesterol uptake and efflux in macrophages.	Hyperlipidemic LDLR^−/−^ mice	[37]
Reduces atherosclerotic plaque area, necrotic core formation and significantly decreases MMP-9 expression associated with plaque rupture, through modulation of macrophage inflammatory signaling pathways (including NF-κB, JAK–STAT, PI3K–Akt and MAPK) and lipid metabolism–related pathways (PPAR signaling), promoting an anti-inflammatory M2 macrophage phenotype	*ApoE*^−/−^ mice	[32]
IL-10 reduces endothelial dysfunction caused by endothelin-1 by improving blood vessel relaxation, increasing eNOS levels, and lowering oxidative stress.	Aortic rings from healthy C57BL/6 mice	[39]
Higher IL-10 levels are linked to lower coronary plaque burden, especially noncalcified plaques, suggesting protective effects against atherosclerosis.	Humans (people with HIV)	[40]

Abbreviations: MMPs—Matrix Metalloproteinases, COX-2—Cyclooxygenase-2, PPARγ—Peroxisome Proliferator-Activated Receptor gamma, LXR—Liver X Receptor, ABCA1—ATP-Binding Cassette Transporter A1, ABCG1—ATP-Binding Cassette Transporter G1, ICAM-1—Intercellular Adhesion Molecule-1, VCAM-1—Vascular Cell Adhesion Molecule-1, eNOS—Endothelial Nitric Oxide Synthase, LDLR^−/−^—Low-Density Lipoprotein Receptor knockout, *ApoE*^−/−^—Apolipoprotein E knockout, Bfl-1—B-cell lymphoma/leukemia 2 family antiapoptotic protein, Mcl-1—Myeloid Cell Leukemia 1 antiapoptotic protein, THP-1 cells—human monocytic leukemia cell line, ACS—Acute Coronary Syndrome, HUVEC—human umbilical vein endothelial cell, JAK1—Janus kinase 1, LOX-1—Lectin-like oxidized low-density lipoprotein receptor-1, MAPK—Mitogen-Activated protein kinase.

**Table 3:** Influence of IL-13 on atherosclerosis development.

Found Peculiarities of IL-13	Cell Type/Mice/Humans	References
IL-13 induces an alternative (M2-like) macrophage phenotype characterized by IL-13 receptor–mediated activation, increased CD36 expression, enhanced oxLDL uptake, and improved cholesterol efflux capacity via upregulation of ABCA1 and ABCG1, resulting in balanced lipid accumulation without net foam cell formation.	IFN-γ–activated macrophages (M1 model, in vitro)	[43]
IL-13 promotes atherosclerotic plaque stabilization by driving alternative (M2-like) macrophage activation, which modulates lesion composition through reduced macrophage accumulation and enhanced fibrotic remodeling, resulting in increased collagen deposition and a shift toward a more stable plaque phenotype without changes in total lesion size.	Atherosclerotic lesions in LDLR^−/−^ mice	[43]
Lower serum IL-13 levels are associated with increased carotid IMT, indicating that IL-13 may exert anti-inflammatory and protective effects in human atherosclerosis.	Older adult human population (>75 years), not selected for a specific disease	[46]

Abbreviations: M2—macrophage with anti-inflammatory phenotype, M1—macrophage with pro-inflammatory phenotype, LDLR^−/−^—Low-Density Lipoprotein Receptor knockout, OxLDL—oxidized low-density lipoprotein, ABCA1—ATP-binding cassette transporter A1, ABCG1—ATP-binding cassette transporter G1, IMT—intima-media thickness, IFN-γ—Interferon gamma.

**Table 4:** Influence of IL-19 on atherosclerosis development.

Found Peculiarities of IL-19	Cell Type/Mice/Humans	References
Acts as a negative feedback cytokine by activating STAT3 in splenic CD11b^+^ macrophages, suppressing pro-inflammatory cytokine production (IL-1β, IL-6, TNF-α, IL-23) and MHC II-mediated antigen presentation, thereby limiting excessive macrophage activation, Th17 expansion, and downstream inflammatory responses.	Splenic CD11b^+^ macrophages from healthy wild type C57BL/6J mice (in vitro cultures and EAE in vivo model)	[52]
IL-19 activates STAT3, upregulates SOCS5 and HO-1, suppresses VSMC proliferation, protects VSMCs from ROS-induced apoptosis, lowers oxidative stress and inflammatory gene expression, and reduces vascular inflammation	Primary human coronary artery vascular smooth muscle cells (VSMCs) in culture	[53]
Reduces aortic plaque size and macrophage infiltration by promoting Th2/Treg polarization, decreasing endothelial VCAM-1, suppressing chemokine and pro-inflammatory cytokine expression (MCP-1, IL-1β, IL-8), and destabilizing pro-inflammatory mRNAs via HuR.	LDLR^−/−^ mice and *ApoE*^−/−^ mice	[54]
IL-19 is expressed in human atherosclerotic plaques, especially in Type IV plaques, with significantly higher levels in symptomatic patients (stroke, TIA, amaurosis fugax). This suggests a counter-regulatory, anti-inflammatory role of IL-19 in modulating vascular inflammation and stabilizing advanced atherosclerotic plaques	Human atherosclerotic plaque tissues from symptomatic and asymptomatic patients, n = 20 per group (coronary and carotid arteries)	[54]

Abbreviations: STAT3—signal Transducter and activator of transcription 3, TNF-α—tumor necrosis factor alpha, MHC II—major histocompatibility complex class II, SOCS5—suppressor of cytokine signaling 5, HO-1—heme oxygenase 1, VSMCs—vascular smooth muscle cells, ROS—reactive oxygen species, VCAM-1—vascular cell adhesion molecule 1, MCP-1—monocyte chemoattractant protein 1, HuR—human antigen R (ELAVL1), LDLR^−/−^ mice—low-density lipoprotein receptor knockout mice, *ApoE*^−/−^ mice—apolipoprotein E knockout mice, Treg—regulatory T cells, TIA—transient ischemic attack.

**Table 8:** Findings about IL-36Ra from cell culture and animal studies.

Found Peculiarities of IL-36Ra	Cell Type/Animal/Human	References
IL-36Ra inhibits IL-36 receptor signaling, suppresses NF-κB/MAPK activation, reduces endothelial inflammation, adhesion molecule expression, and limits leukocyte recruitment; acts as a natural blocker of IL-36α/β/γ signaling, like IL-1Ra.	Endothelial cells (HUVECs), epithelial cells, monocytes, macrophages, and other immune cells.	[92,93]
IL-36Ra antagonizes IL-36 signaling by binding IL-36R and preventing IL-1RAcP recruitment, suppresses NLRP3 inflammasome activation, reduces neutrophil recruitment, endothelial oxidative damage and inflammation in cardiovascular models.	Endothelial cells/*ApoE*^−/−^ mice (atherosclerosis, ischemia-reperfusion injury models).	[93]
IL-36 promotes coronary microvascular inflammation, whereas IL-36Ra reduces inflammation and microvascular damage in myocardial ischemia-reperfusion injury in mice. Treatment with IL-36Ra reduces neutrophil recruitment, endothelial oxidative stress, VCAM-1 expression, improves coronary perfusion, and decreases infarct size in both sexes, despite sex-specific differences in neutrophil/platelet responses.	Coronary endothelial cells, cardiomyocytes/Adult and aged mice (myocardial ischemia–reperfusion injury).	[94]

Abbreviations: NF-κB—nuclear factor kappa B, MAPK—mitogen-activated protein kinase.

**Table 9:** Main findings of IL-38 role in atherosclerosis from cell culture, mice and human studies.

Found Peculiarities of IL-38	Cell Type/Mice/Humans	References
Inhibition of macrophage M1-like polarization and apoptosis and reduced secretion of IL-1β, IL-6, TNF-α.	Cultured macrophages.	[103]
Reduction of atherosclerotic plaque area and vascular inflammation.	Hyperlipidemic mice.	
IL-38 attenuates vascular calcification.	VSMCs and macrophages.	[104]
Attenuation of vascular calcification via antioxidant mechanisms.	Mice models of vascular calcification.	
IL-38 promotes SIRT6 and HO-1 expression; IL-38 alleviates inflammation via SIRT6/HO-1 pathway, thereby improving atherogenic responses.	HUVECs and THP-1 monocytes	[101]
Reduced inflammatory damage after myocardial ischemia.	Murine ischemia models.	[102]
Reduced IL-38 expression in human calcified coronary tissues, patient plasma, and experimental models; IL-38 activates the PPAR-γ/NRF2 signaling axis to regulate GPX3 expression; results identify IL-38 as a novel endogenous inhibitor of vascular calcification operating through the PPAR-γ/NRF2/GPX3 pathway, providing mechanistic insight and suggesting a potential therapeutic strategy.	Mice, cell culture hASMCs, coronary artery tissues of patients with CAC (n = 6), and without CAC (n = 6).	[104]
Activation of SIRT6/HO-1 signaling and inhibition of inflammatory gene expression.	Macrophages.	[105]
Suppression of obesity-related atherogenic responses through SIRT6/HO-1 signaling.	High-fat diet-fed mice.	[105]
Circulating IL-38 is detectable and stable over time; levels decrease with age	288 healthy individuals, 119 male and 169 female	[106]
Reduced circulating IL-38 is associated with chronic inflammation	296 Overweight individuals with increased cardiovascular risk (BMI > 27), including subjects with metabolic syndrome	[106]
Predictive value of IL-38 for adverse cardiovascular events after PCI.	CAD patients after PCI. 408 patients, 194 patients had MACE and 214 MACE-free.	[107]

Abbreviations: GPX3—glutathione peroxidase 3, VSMCs—Vascular Smooth Muscle Cells, CAD—Coronary Artery Disease, PCI—Percutaneous Coronary Intervention, CVD—Cardiovascular disease, MACE—Major adverse cardiovascular events, BMI—body mass index, hASMCs—Primary human aortic smooth muscle cells, GPX3—glutathione peroxidase 3, PPAR-γ—Peroxisome Proliferator-Activated Receptor γ; NRF2—Nuclear factor erythroid 2-related factor 2, HUVECs—Human umbilical vein endothelial cells, THP-1—a human leukemia monocytic cell line; CAC—with coronary artery calcification, SIRT6—sirtuin 6.

**Table 10:** Summarizing knowledge about Anti-inflammatory ILs actions on cells, receptor usage, downstream signaling and what still needs to be clarified.

IL/References	Cellular Sources	Receptor Complexes for IL Action	Signaling Cascades	Effects on Blood Cells	Effects on Cells in the Arterial Walls	Influence on Lipid Metabolism and Foam Cell Formation	What Still Needs to Be Clarified
IL-5/[13,14,16,21]	ILC2, Th2 lymphocytes.	IL-5R (subunits IL-5Rα and βc)	IL-33/IL-25/TSLP → ILC2 → IL-5 → IgM pathway, ABCA1-mediated pathway.	Stimulates the differentiation of B and T cells, stimulates ABCA1 and cholesterol efflux in THP-1-derived macrophages.	Limits the apoptosis of smooth muscle cells.	Mediated cholesterol efflux through the miR-211/JAK2/STAT3 signaling pathway in THP-1-derived macrophages. Inhibition of foam cell formation.	Exact molecular pathways by which IL-5 regulates ABCA1/ABCG; Whether IL-5 effects are direct or mediated via other cells; Effects of different IL-5 SNPs on atherosclerosis; Clinical relevance in different patient populations.
IL-10/[28,36,108,109]	Regulatory T cells, macrophages, B cells.	IL-10R1/IL-10R2.	JAK1/TYK2 → STAT3 activation. IL-10 modulates vascular inflammation by regulating RhoA/Rho kinase signaling.	Inhibits activation of macrophages and suppresses the production of pro-inflammatory cytokines such as TNF-α, IL-1β, IL-6, and IL-8, granulocyte stimulating factor (G-CSF) and granulocyte macrophage colony-stimulating factor (GM-CSF).	Suppresses VSMC proliferation and reduces MMP expression, thereby limiting vascular remodeling and plaque destabilization.	Increases uptake of modified LDL and foam cell formation by upregulating scavenger receptors (CD36, SR-A), while also promoting cholesterol efflux through PPARγ, ABCA1 pathways; induces expression of the antiapoptotic genes Bfl-1 and Mcl-1 in macrophages, which inhibits oxLDL-induced apoptosis of lipid-laden foam cells. By preserving foam cell survival, IL-10 contributes to the stabilization of atherosclerotic plaques, reduces necrotic core. Reduces cholesterol accumulation in the vascular wall via LOX-1–mediated mechanisms.of formation and the risk of plaque rupture.	Patient specific effects; precise modulation in atherosclerotic plaques; crosstalk with other cytokines in human CVD.
IL-13/[43]	Th2 lymphocytes, CD4^+^ T, T cells, mast cells, NK cells, basophils, and eosinophils	Type II IL-13 receptor (IL-4Rα subunit + IL-13Rα1 chain), IL-13Rα2 receptor	IL-13 → IL-13Rα1/IL-4Rα pathway activates JAK1/JAK3 and STAT6 signaling, leading to STAT6 phosphorylation and nuclear translocation IL-13 → IL-13Rα2 pathway inhibits IL-13 signaling by acting as a decoy receptor and limiting STAT6 activation.	Promotes M2 polarization and suppresses pro-inflammatory cytokine production such as TNF-α, IL-1β, and IL-6 in macrophages; inhibits classical (M1) macrophage activation and reduces NF-κB–mediated inflammatory responses.	Reduces vascular inflammation by inhibiting activation of macrophages within atherosclerotic plaques; decreases expression of inflammatory mediators and reduced the recruitment and adhesion of monocytes to the atherosclerotic wall; IL-13 promotes a repair-oriented (M2) macrophage phenotype that supports tissue remodeling by enhancing collagen synthesis and extracellular matrix formation.	Stimulates M2 macrophages to uptake oxidized LDL more efficiently than M1 macrophages, enhance cholesterol efflux through ABC transporters (ABCA1, ABCG1), preventing excessive foam cell formation.	The differential effects of IL-13 compared to IL-4 on vascular cells remain unclear due to the lack of direct comparisons in the same disease models, and the specific contributions of IL-13 receptors, particularly IL-13Rα2, to vascular protection, collagen deposition, plaque stability, and their relevance in humans require further investigation.
IL-19/[54,110]	Lipopolysaccharide (LPS)-stimulated monocytes, keratinocytes, endothelial cells.	IL-20Rα/IL-20Rβ receptor complex	IL-19 binds IL-20Rα/IL-20Rβ → activates JAK-STAT3/STAT6 pathways	IL-19 promotes Th2 polarization and reduces pro-inflammatory cytokine expression in immune cells by decreasing Th1 cytokines (such as IFN-γ, IL-1β and IL-12β) and increasing Th2 markers like GATA3 and FoxP3.	IL-19 treatment markedly decreases macrophage infiltration in atherosclerotic plaques and lowers leukocyte–endothelial adhesion by decreasing monocyte adhesion to the vessel wall through inhibition of VCAM-1.	IL-19 helps control macrophage cholesterol balance by upregulating scavenger receptors (e.g., CD36, SRA-1, SR-B1) and promoting both oxidized LDL uptake and cholesterol efflux through ABC transporters (ABCA1, ABCG1) in a PPARγ-dependent way, without notably increasing macrophage apoptosis.	The specific molecular mechanisms of IL-19 in vascular cells, how IL-20Rα/IL-20Rβ receptor signaling through JAK-STAT3/STAT6 translates into atheroprotection, receptor specificity, integration with lipid metabolism, oxidative stress, immune cell polarization in atherosclerotic lesions, and therapeutic potential in humans remain poorly understood.
IL-35/[57,59,111]	Regulatory T cells, regulatory B cells, monocytes, endothelial cells, SMC, epithelial cells	IL-12Rβ2 and gp130 receptor complex	IL-35 activates STAT1, STAT3 and STAT4 signaling in endothelial cells. Signaling pathways involve miRNR.	Enhances Treg activity and suppresses effector T cell responses, promoting an anti-inflammatory immune profile.	IL-35 inhibits endothelial cell activation by inhibiting mtROS generation and reducing the expression of adhesion molecules such as VCAM-1 and ICAM-1, which leads to decreased monocyte adhesion to the endothelium and reduced vascular inflammation.	IL-35 reduces foam cell formation in oxLDL-stimulated macrophages by lowering lipid accumulation and modulating CAD-associated miRNAs that regulate macrophage polarization, lipid metabolism, and inflammation, resulting in a protective effect against atherosclerosis.	Complete molecular mechanism in atherosclerosis; receptor expression in human plaques. The molecular mechanisms of action and relevance in humans. Future studies also need to address whether IL-35 specifically modulate some, if not all, of mtROS regulating factors to inhibit mtROS; and whether other anti-inflammatory cytokines, such as IL-10 and TGF-β, could inhibit mtROS. The roles of its subunits EBI3 and p35, the regulation of IL-35 expression under pro-atherogenic stimuli, require further investigation. The precise mechanisms by which IL-35 modulate endothelial function, foam cell formation, and macrophage polarization in atherosclerotic lesions remain unclear.
IL-1Ra/[79,112]	Fibroblasts, monocytes, mast cells, neutrophils, B cells, T cells, macrophages, endothelial cells, epithelial cells	IL-1 type I receptor (IL-1RI)	Binds IL-1RI → blocks IL-1α/IL-1β signaling → inhibits NF-κB and MAPK	Monocytes/macrophages reduces IL-1β amplification lowers TNF-α and IL-6 production decreases inflammatory activation Neutrophils reduces recruitment and activation in inflamed tissues T cells indirectly suppresses Th1 and Th17 responses supports a more regulated immune profile.	Endothelial cells reduces expression of VCAM-1, ICAM-1 decreases leukocyte adhesion and transmigration VSMCs inhibits proliferation and inflammatory activation reduces cytokine-induced remodeling Macrophages in plagues reduces foam cell inflammatory activity.	IL-1Ra does not directly regulate lipid metabolism, but it: reduces inflammation-driven lipid uptake in macrophages decreases oxLDL-induced foam cell formation indirectly improves systemic inflammatory lipid dysregulation By blocking IL-1 signaling: reduces endothelial dysfunction. improves cholesterol handling in inflamed vessels.	The differences between secretory and intracellular IL-1Ra forms, the molecular functions of intracellular isoforms, their effects in humans, therapeutic applications, and whether circulating IL-1Ra levels directly reflect early atherosclerotic changes or systemic inflammation remain unclear.
IL-36Ra/[113]	Dendritic cells, epithelial cells, endothelial cells, fibroblasts, keratinocytes, monocytes, macrophages	IL-36 receptor (IL-36R)	Binds IL-36R → inhibits IL-36α/β/γ signaling	Macrophages reduces production of IL-6, TNF-α, IL-1β limits inflammatory activation Dendritic cells decreases maturation and antigen-presenting activity T cells indirectly reduces Th1 and Th17 responses.	Endothelial cells reduces expression of adhesion molecules decreases leukocyte recruitment VSMCs inhibits inflammatory activation may reduce proliferation and migration.	Indirectly reduces foam cell formation by suppressing inflammation may limit macrophage activation and lipid uptake By antagonizing IL-36 signaling: reduces pro-atherogenic environment may slow plaque progression.	The precise receptor interactions, cellular targets, molecular mechanisms, effects in human atherosclerosis, and therapeutic relevance of IL-36Ra remain insufficiently defined, and further studies are needed to clarify its functional role in cardiovascular disease and metabolic inflammation.
IL-37/[66,67,114]	Monocytes, macrophages, dendritic cells, epithelial cells, endothelial cells, fibroblasts, SMC, some tumor cells	IL-1R8 (orphan receptor)	Intracellular Smad3/extracellular IL-1R8 signaling	Monocytes/macrophages decreased production of IL-1β, IL-6, TNF-α reduced inflammatory activation Macrophage polarization shifts toward M2 (anti-inflammatory phenotype) T cells suppresses Th1 and Th17 responses enhances regulatory T cell (Treg) activity.	Endothelial cells reduces expression of adhesion molecules (e.g., VCAM-1, ICAM-1) decreases leukocyte adhesion VSMCs inhibits proliferation and migration reduces inflammatory activation Also: decreases plaque inflammation promotes plaque stability	Improves lipid metabolism reduces cholesterol accumulation in macrophages Mechanisms: enhances cholesterol efflux (e.g., via ABCA1 pathways) reduces uptake of modified LDL Result: decreased foam cell formation attenuation of atherosclerotic plaque development	Mechanisms in different patient populations; therapeutic potential in atherosclerosis. How Smad3 and IL-1R8 signaling interact, the detailed receptor complexes, and the role of IL-37 in various cardiovascular pathologies in humans.
IL-38/[96,115]	B cells, macrophages, cardiomyocytes.	IL-1R1, IL-36R, IL1RAPL1 (TIGIRR-2).	IL-1R1/IL-36/IL1RAPL1 –MyD88–NF-κB pathway; MAPK pathway (p38/ERK1/2/JNK inhibition); NLRP3 inflammasome inhibition; IL1RAPL1 (TIGIRR-2)–associated inhibitory signaling.	Macrophages inhibits M1 (pro-inflammatory) polarization promotes M2 (anti-inflammatory) phenotype reduces secretion of IL-1β, TNF-α T cells modulates the balance between Th1, Th17, and Treg cells contributes to immune suppression and resolution of inflammation. Result: decreased systemic and vascular inflammation.	IL-38 indirectly affects: endothelial cells, Vascular smooth muscle cells (VSMCs) Observed effects: reduced inflammatory activation inhibition of apoptosis within plaques decreased pathological neovascularization	Modulates cholesterol metabolism in macrophages reduces oxLDL-induced activation It indirectly limits foam cell formation by: decreasing M1 polarization suppressing inflammatory signaling Additionally: may improve hyperlipidemia and metabolic risk factors. IL-38 limits the size and number of adipocytes and reduces the accumulation of lipids in atherosclerotic lesions	The exact receptor combinations responsible for IL-38 signaling, including the roles of IL-1R1, IL-36R, and IL1RAPL1, remain to be specified. The detailed mechanisms underlying IL-38–mediated inhibition of NF-κB, MAPK, and NLRP3 pathways, as well as its therapeutic relevance and optimal recombinant form, require further investigation.

Abbreviations: ABCA1—ATP-binding cassette transporter A1, ABCG1—ATP-binding cassette transporter G1, CAD—coronary artery disease, CD36—cluster of differentiation 36, ERK1/2—extracellular signal-regulated kinase 1/2, FoxP3—forked box P3, G-CSF—granulocyte colony-stimulating factor, GATA3—GATA binding protein 3, GM-CSF—granulocyte–macrophage colony-stimulating factor, ICAM-1—intercellular adhesion molecule 1, IFN-γ—interferon gamma, IL1RAPL1—interleukin-1 receptor accessory protein-like 1, ILC2—type 2 innate lymphoid cells, JAK—Janus kinase, JNK—c-Jun N-terminal kinase, LDL—low-density lipoprotein, M1—macrophage with pro-inflammatory phenotype, M2—macrophage with anti-inflammatory phenotype, MAPK—mitogen-activated protein kinase, miRNA (miR)—microRNA, MMP—matrix metalloproteinase, mtROS—mitochondrial reactive oxygen species, MyD88—myeloid differentiation primary response 88, NF-κB—nuclear factor kappa B, NLRP3—NOD-like receptor family pyrin domain containing 3, ox-LDL—oxidized low-density lipoprotein, PPARγ—peroxisome proliferator-activated receptor gamma, Smad3—SMAD family member 3, SNP—single nucleotide polymorphism, SR-A—scavenger receptor class A, SR-B1—scavenger receptor class B type 1, SRA-1—scavenger receptor A1, STAT—signal transducer and activator of transcription, THP-1—human acute monocytic leukemia cell line, TYK—tyrosine kinase, VCAM-1—vascular cell adhesion molecule 1, VSMC—vascular smooth muscle cell.

## Data Availability

Not applicable.
